# Synthesis and Antimalarial Evaluation of Halogenated Analogues of Thiaplakortone A

**DOI:** 10.3390/md21050317

**Published:** 2023-05-22

**Authors:** Folake A. Egbewande, Brett D. Schwartz, Sandra Duffy, Vicky M. Avery, Rohan A. Davis

**Affiliations:** 1 Griffith Institute for Drug Discovery, School of Environment and Science, Griffith University, Nathan, QLD 4111, Australia; f.egbewande@griffith.edu.au (F.A.E.); b.schwartz@griffith.edu.au (B.D.S.); sandra.duffy@griffith.edu.au (S.D.); v.avery@griffith.edu.au (V.M.A.); 2 Discovery Biology, Centre for Cellular Phenomics, Griffith University, Nathan, QLD 4111, Australia; 3 NatureBank, Griffith University, Nathan, QLD 4111, Australia

**Keywords:** halogenation, late-stage functionalization, *N*-bromosuccinimide, *N*-iodosuccinimide, sulfinate, Diversinate™, natural product, thiaplakortone A, library, malaria, *Plasmodium*, biodiscovery

## Abstract

The incorporation of bromine, iodine or fluorine into the tricyclic core structure of thiaplakortone A (**1**), a potent antimalarial marine natural product, is reported. Although yields were low, it was possible to synthesise a small nine-membered library using the previously synthesised Boc-protected thiaplakortone A (**2**) as a scaffold for late-stage functionalisation. The new thiaplakortone A analogues (**3**–**11**) were generated using *N*-bromosuccinimide, *N*-iodosuccinimide or a Diversinate™ reagent. The chemical structures of all new analogues were fully characterised by 1D/2D NMR, UV, IR and MS data analyses. All compounds were evaluated for their antimalarial activity against *Plasmodium falciparum* 3D7 (drug-sensitive) and Dd2 (drug-resistant) strains. Incorporation of halogens at positions 2 and 7 of the thiaplakortone A scaffold was shown to reduce antimalarial activity compared to the natural product. Of the new compounds, the mono-brominated analogue (compound **5**) displayed the best antimalarial activity with IC_50_ values of 0.559 and 0.058 μM against *P. falciparum* 3D7 and Dd2, respectively, with minimal toxicity against a human cell line (HEK293) observed at 80 μM. Of note, the majority of the halogenated compounds showed greater efficacy against the *P. falciparum* drug-resistant strain.

## 1. Introduction

Malaria is one of the world’s most severe infectious diseases caused by a protozoan of the genus *Plasmodium*; around 50% of the world’s population are at risk of contracting this debilitating and deadly disease [1,2]. The World Health Organization (WHO) World Malaria Report in 2022 estimated that 619,000 deaths and 245 million cases of malaria occurred globally during 2021, a slight decrease (~1%) from the previous year [2]. Disruptions to essential malaria services during the COVID-19 pandemic between 2019 and 2021 are believed to have contributed to 63,000 deaths [2]. The WHO recommends key intervention measures that include vector control using insecticide-treated mosquito nets (ITNs) and indoor residual spraying (IRS), and artemisinin-based combination therapy (ACT). About 663 million cases have been prevented in sub-Saharan Africa since 2001 as a result of these interventions [2]. However, parasite resistance to artemisinin and derivatives has been confirmed in the Greater Mekong subregion of Southeast Asia. Additionally, poor safety profiles and unwanted side-effects associated with known antimalarials necessitate the need for new small molecule therapeutics with high selectivity towards the malaria parasite [3,4,5,6]. Therefore, it is imperative that new antimalarial drugs that are effective through alternative mechanisms of action to those currently in use are developed to combat this globally devastating disease.

**Figure 1:**
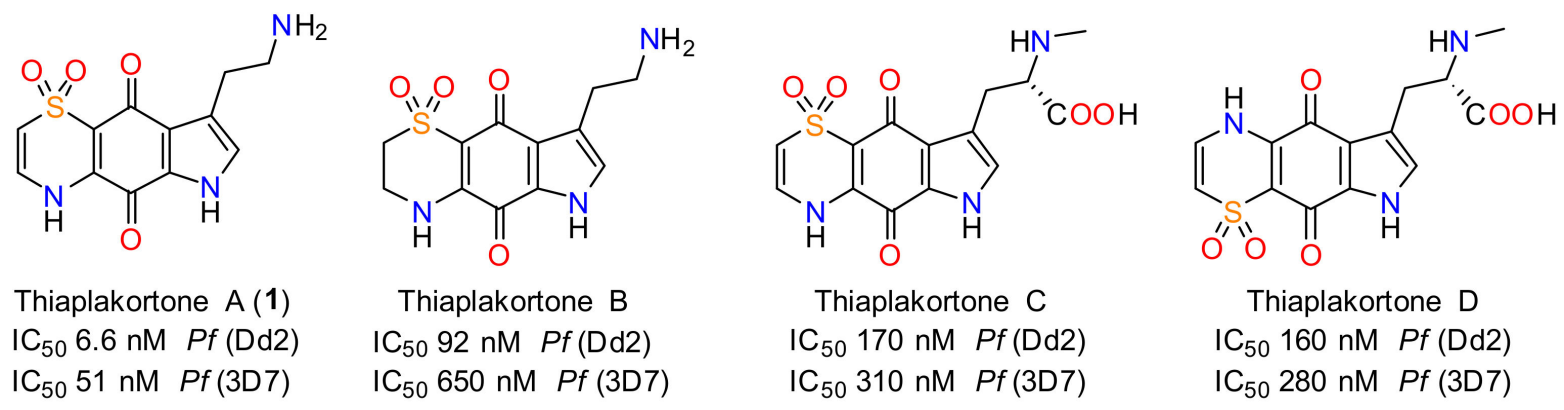
Chemical structures of thiaplakortones A–D and in vitro antimalarial activity [9].

With the intent of discovering new antimalarials from nature, the NatureBank fraction library (202,983 fractions) was screened during 2007 against *Plasmodium falciparum* 3D7 with one of the identified hit fractions derived from the Australian marine sponge *Plakortis lita* [7,8]. Subsequent bioassay-guided fractionation of the sponge extract resulted in the isolation of four novel thiazine-derived alkaloids that were given the trivial names thiaplakortones A–D (see Figure 1) [9]. These compounds were screened against drug-sensitive (3D7) and drug-resistant (Dd2) *P. falciparum* parasites, and cytotoxicity data were also acquired using the human embryonic cell line HEK293. Thiaplakortone A (**1**) was the most active compound with IC_50_ values of 6.6 nM and 51 nM against Dd2 and 3D7 *P. falciparum* parasites, respectively. Furthermore, natural product **1** showed low cytotoxicity against HEK293 cells, which translated to selectivity indices of 591 and 76 against the Dd2 and 3D7 strains, respectively [9].

In an effort to generate lead compound(s) with improved activity and absorption, distribution, metabolism, excretion, and toxicity (ADMET) properties, various analogues based on the thiaplakortone A (**1**) natural product scaffold (e.g., amides and ureas) were synthesised and evaluated for their antimalarial activity, with several of the analogues showing potent inhibition of *P. falciparum* growth and better metabolic stability than the lead natural product [10,11]. However, poor oral bioavailability and short half-lives associated with some of the more promising leads limited further development.

To further explore the structure-activity relationships (SARs) for this rare class of marine-derived natural compound, herein we report a small late-stage functionalisation study that generated and characterised several new halogenated thiaplakortone A analogues.

## 2. Results and Discussion

Since the primary amine functionality of thiaplakortone A (**1**) had been previously exploited to generate >30 amide and urea analogues and map SAR around this portion of the molecule [10,11], we decided at the outset of this study to make new structural modifications around the tricyclic system. Specifically, the incorporation of halogen atoms into the tricyclic core of compound **1** was targeted. The insertion of halogen atoms into bioactive compounds has been a widely employed strategy for transforming hits and/or leads into potential drugs [12,13,14,15]. To enhance oral absorption, halogen atom insertion is frequently utilised to increase the permeability of bioactive compounds across the blood-brain barrier and cell membranes [16,17]. Furthermore, the incorporation of halogen atoms into hit/lead compounds is also performed to exploit their steric effects through the ability of these bulky atoms to occupy the active site of molecular targets [18,19,20] and establish intermolecular X-bonds in a manner that resembles H-bonds [21,22]. Gunaydin and co-workers showed that the half-life of a compound could be improved by the introduction of halogens and that the extent to which the half-life increases is proportional to the number of halogens added to such molecules [23].

**Scheme 1:**
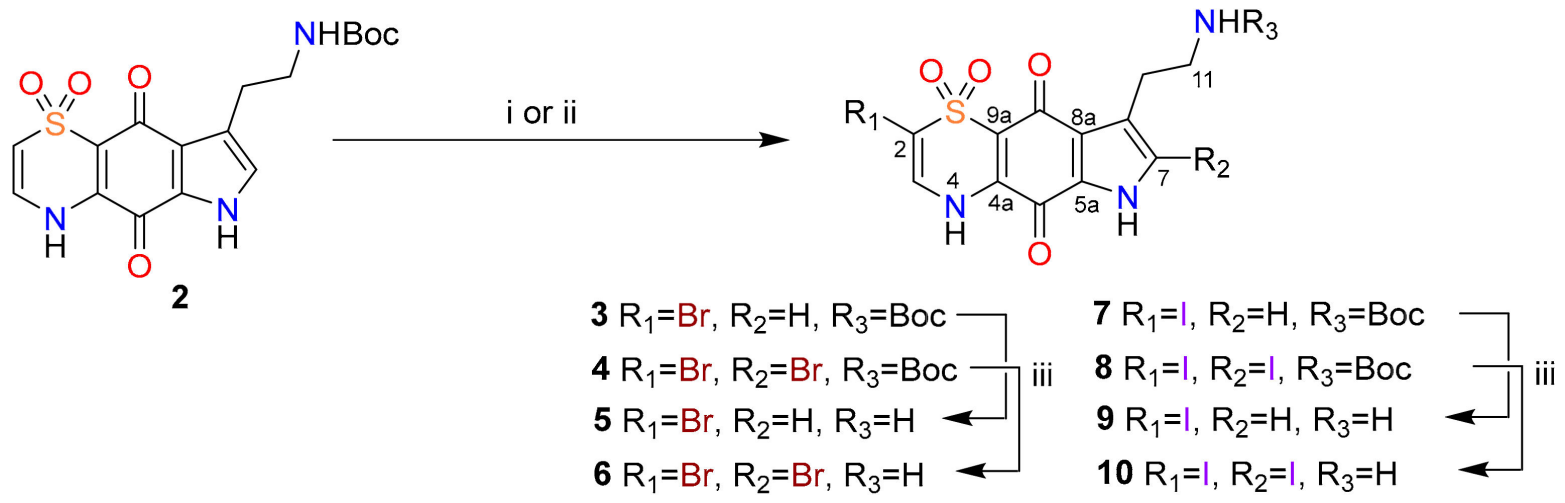
(i) NBS, CH_3_CN, 0 °C to room temperature, 16 h; (ii) NIS, CH_3_CN, 0 °C to room temperature, 16 h; (iii) CH_2_Cl_2_/TFA, rt, 1 h.

Thiaplakortone A (and intermediates) was produced via total synthesis as previously described [10,11]; this earlier synthetic work enabled the halogenation investigations reported here to be undertaken (see Scheme 1). The first halogenated thiaplakortone A derivative made during the current studies used the versatile reagent, *N*-bromosuccinimide (NBS). Whilst numerous methods for halogenation of pyrroles have been reported, a procedure reported by Khan et al. that employed NBS for the late-stage bromination of the natural product breitfussin B appealed to us due to the operationally simple nature and mild reaction conditions of this transformation [24]. Accordingly, we exposed Boc-protected thiaplakortone A (**2**) to a solution of NBS in CH_3_CN, and under these conditions, the 2-bromo analogue **3** was obtained in 9% yield along with the 2,7-dibromo derivative **4** (33% yield). Subsequent Boc removal of these brominated analogues under standard TFA-mediated deprotection conditions [10] afforded the TFA salts of **5** and **6**.

The analogous iodinated derivatives, **7** and **8** were similarly obtained by employing *N*-iodosuccinimide (NIS) in place of NBS. Yields for the iodinated products were also poor (<8%), and the iodination reaction had to be repeated several times in order to provide sufficient quantities (~5 mg) of **7** and **8** for Boc deprotection and generation of the iodinated analogues **9** and **10**. As with the bromination reaction, iodination was selective for the C-2 and C-7 positions. An attack of the electrophilic halogen at C-2 rather than C-3 is to be expected, as this is analogous to the bromination of an enamine. C-7 halogenation could occur via either a radical or electrophilic pathway. In the simplified 3-methylindole system, Cook et al. suggested an electrophilic bromination pathway operated in the absence of an electron-withdrawing protecting group on the indole nitrogen [25]. However, due to the complexity of the thiaplakortone A scaffold, it is difficult to determine a precise mechanism that explains this selectivity.

**Figure 2:**
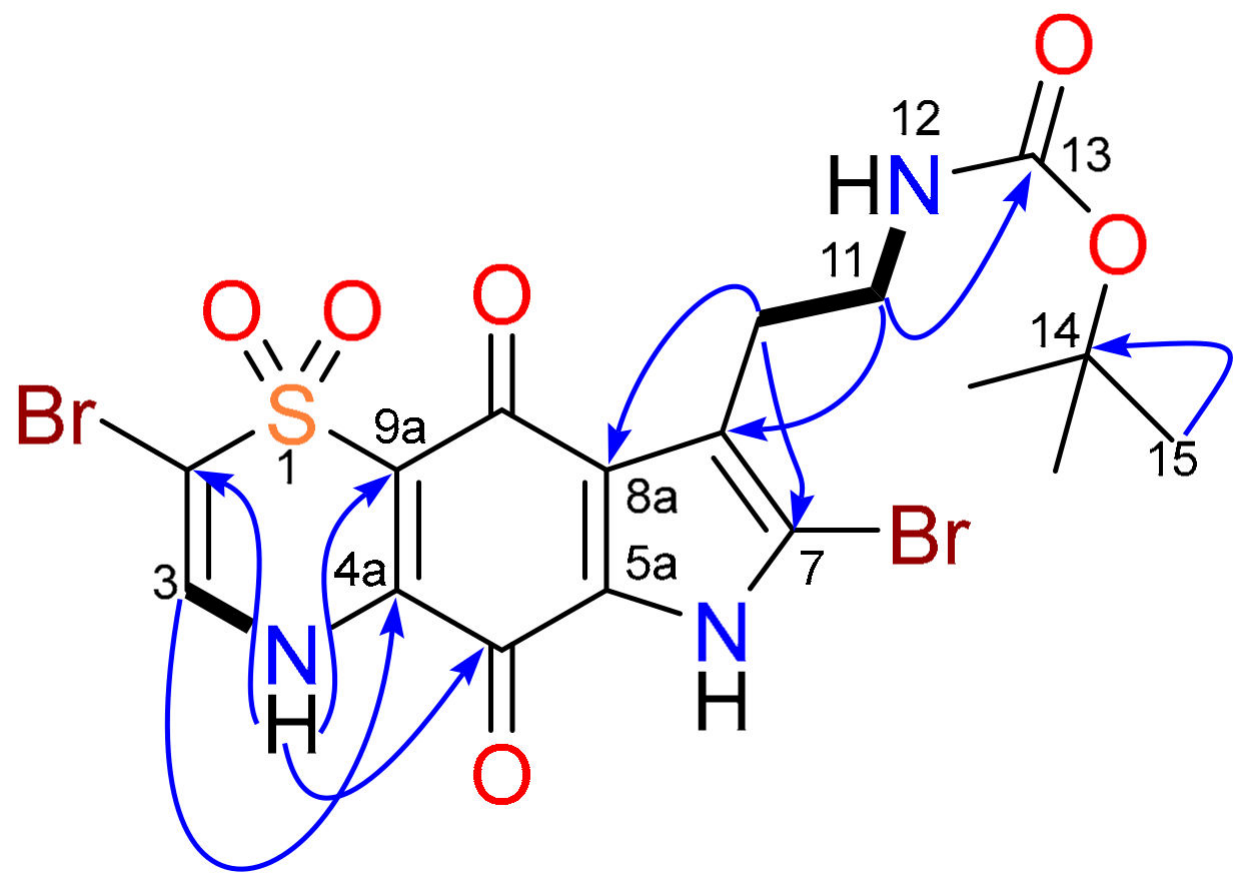
COSY (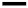) and key HMBC (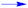) correlations for compound **4**.

All halogenated products **3**–**10** were purified by C_18_-bonded silica reversed-phase HPLC (MeOH/H_2_O/0.1% TFA) to yield high-purity materials (>95% purity). The chemical structures of all halogenated analogues were determined by 1D/2D NMR and MS data analyses. An example of the structure confirmation and NMR characterisation of compound **4** is described below. Briefly, the ^1^H NMR spectrum in DMSO-*d*_6_ for **4** (Supplementary Materials) indicated the presence of nine methyl protons [*δ*_H_ 1.35 (H-14)], two methylenes [*δ*_H_ 2.74 (H-10), and 3.08 (H-11)], one aromatic proton [*δ*_H_ 7.41 (H-3)], and three exchangeable protons [*δ*_H_ 6.80 (H-12), δ_H_ 11.38 (H-4), and 13.91(H-6)] and the (−)-LRESIMS showed a 1:2:1 ion cluster at *m*/*z* 548:550:552; these data were consistent for a dibrominated *N*-Boc thiaplakortone A derivative. While analysis of the COSY spectrum revealed the presence of two spin systems, the interpretation of the HMBC data definitively assigned the substitution of the two bromine atoms present in compound **4** (see Figure 2). Several key HMBC correlations, especially those associated with methylene protons found in the ethylamine side chain [*δ*_H_ 2.74 (H-10), and 3.08 (H-11)] and the thiazine methine and exchangeable NH protons [*δ*_H_ 7.41 (H-3) and *δ*_H_ 11.38 (H-4)] were crucial for placement of the bromines in **4**.

**Scheme 2:**
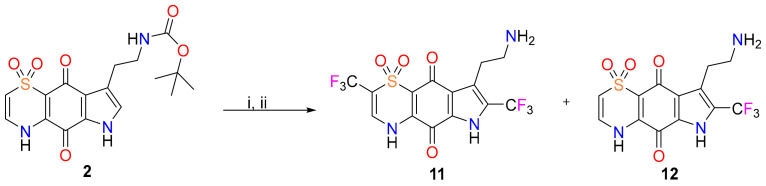
(i) Zinc trifluoromethanesulfinate, CH_2_Cl_2_/H_2_O, 4 °C, 1 h; (ii) *tert*-butyl hydroperoxide, 4 °C to room temperature, 16 h.

To further expand our library and thus explore SARs within the halogenated series, we sought to engage Baran’s innovative Diversinate™ chemistry for the late-stage trifluoromethylation of *N*-Boc protected thiaplakortone A [26,27]. Consequently, a mixture of compound **2** and zinc trifluoromethanesulfinate in CH_2_Cl_2_/H_2_O was stirred for 1 h at 4 °C (see Scheme 2). Aqueous *tert*-butyl hydroperoxide (TBHP) was added over 5 min, and the mixture was slowly warmed to room temperature over 16 h with stirring. The reaction products were purified using C_18_ HPLC (MeOH/H_2_O/0.1% TFA) to yield the di-trifluoromethyl derivative **11** in low yield (15%) but high purity (>95%). In addition, a small amount of a mono-trifluoromethyl derivative (potentially **12**) was also tentatively identified following analysis by MS and ^1^H NMR spectroscopy of one C_18_ HPLC fraction, albeit as an enriched mixture. However, limited amounts of this material prevented additional purification work and due to a lack of full 1D/2D NMR studies, the chemical structure of **12** was not able to be definitively assigned.

In a similar manner to the brominated and iodinated analogues (**3**–**10**) detailed in this study, 1D/2D NMR and MS data analysis confirmed the structure for **11** with trifluoromethylation at C-2 and C-7 preferred. These encouraging data suggested further Diversinate™ chemistry may be warranted on the thiaplakortone A scaffold, although some future optimisation of this particular chemistry is required to improve yields.

Prior to the biological evaluation, we analysed compounds **1**–**11** using ChemAxon’s online prediction software Chemicalize [28] and calculated in silico physicochemical parameters (as shown in Table 1), which were then compared to Lipinski’s “Rule of Five” (Ro5) for drug-like compounds [29]. All compounds except **7**, **8** and **10** met the criteria of Lipinski’s rules, with cLog P values less than 5, less than 10 hydrogen bond acceptors (HBA), less than 5 hydrogen bond donors (HBD), and a molecular weight (MW) of less than 500 Da [29]. Compounds **7**, **8** and **10** only had one Ro5 violation, with their MW values all greater than 500 Da, due to their incorporation of iodine. Additionally, all compounds were predicted to have suitably low cLog D_7.4_ values for further preclinical drug development [30].

Compounds **1**–**11** were evaluated in vitro for their ability to inhibit the proliferation of two strains of the malaria parasite, drug-sensitive 3D7 and multidrug-resistant Dd2 (see Table 2). In addition, compounds were also tested for potential cytotoxicity using human embryonic kidney cells (HEK293). A noteworthy observation following analysis of the screening data is that, like the thiaplakortone natural products [8], the halogenated compounds are more active against the drug-resistant Dd2 parasite line compared to the drug-sensitive line. Out of all the new analogues (**3**–**11**) generated during these studies, the mono-brominated and Boc deprotected derivative **5** was shown to be the most active with IC_50_ values of 0.559 and 0.058 μM against *P. falciparum* 3D7 and Dd2, respectively, with minimal cytotoxicity against HEK293 cells observed at 80 μM. Replacing the bromine at C-2 in compound **5** with iodine (i.e., compound **9**) reduced the antimalarial activity against 3D7 and Dd2 strains by 3.9- and 5.5-fold, respectively. These data indicated that the steric bulk of iodine compared to bromine at C-2 was detrimental to antimalarial activity. The next most active new derivative, the dibrominated and Boc-protected compound **4** (IC_50_ values of 1.373 and 2.060 μM against *P. falciparum* 3D7 and Dd2, respectively), showed a marginal decrease (1.2-fold) in antimalarial activity against the 3D7 strain upon removal of the Boc group; however, the activity against the Dd2 strain was improved (3.1-fold). Finally, an increase of antimalarial activity (2.5-fold for 3D7; 3.7-fold for Dd2) was noted when the *N*-Boc group of mono-iodinated compound **7** was removed to generate compound **9**. Furthermore, compounds **9** and **10** had similar 3D7 and Dd2 activity, suggesting that di-iodination had minimal impact on the antimalarial activity.

## 3. Materials and Methods

### 3.1. Chemistry Procedures

UV spectra were recorded using a JASCO V-650 UV/vis spectrophotometer (Easton, MD, USA). IR data were acquired on a Universal Attenuated Total Reflectance (UATR) two-module attachment on a PerkinElmer spectrophotometer (Waltham, MA, USA). NMR spectra were recorded at 25 °C on a Bruker Avance III HD 800 MHz spectrometer equipped with a cryoprobe (Bruker, Billerica, MA, USA). The ^1^H and ^13^C NMR chemical shifts were referenced to the solvent peak for DMSO-*d*_6_ at *δ*_H_ 2.50 and *δ*_C_ 39.5. LRESIMS data were recorded on an Ultimate 3000 RS UHPLC coupled to a Thermo Fisher Scientific ISQEC single quadruple ESI mass spectrometer using an analytical Thermo Scientific Accucore C_18_ (2.6 μm, 80 Å, 150 × 2.1 mm) (Thermo Fisher Scientific, Waltham, MA, USA). HRESIMS data were recorded on 12 T Bruker SolariX XR FT-ICR-MS (Bruker, Billerica, MA, USA). A Dionex Ultimate 3000 HPLC was used for semi-preparative HPLC separations (Thermo Fisher Scientific, Waltham, MA, USA). Grace Davisil C_18_ bonded silica (35–75 µm, 150 Å; Columbia, MD, USA) was used for pre-adsorption of the crude reaction products before HPLC separations. A Grace stainless steel guard cartridge (10 mm × 30 mm; Columbia, MD, USA) was used for loading pre-adsorbed synthetic reaction products onto the semi-preparative HPLC columns. A Phenomenex Luna C_18_ column (5 µm, 90–110 Å, 10 mm × 250 mm; Torrance, CA, USA) or a Thermo Electron Betasil C_18_ column (5 µm, 100 Å, 21.2 mm × 150 mm; Thermo Fisher Scientific, Waltham, MA, USA) was used for semi-preparative HPLC separations. All solvents used for chromatography were Lab-Scan HPLC grade (ChemSupply, Port Adelaide, SA, Australia), and the H_2_O was Sartorius Arium Pro VF ultrapure filtered (Sartorius, Göttingen, Germany). All synthetic reagents were purchased from Sigma-Aldrich (Castle Hill, NSW, Australia) and used without further purification. ChemDraw Ultra 12.0.2 software was used to draw all chemical structures and analyse physicochemical parameters. MestReNova 11.0.4 software was used for NMR data analysis.

### 3.2. Halogenation Reactions

A magnetically stirred solution of *N*-Boc protected thiaplakortone A (**2**, 25.0 mg, 0.063 mmol) in CH_3_CN (1 mL), maintained in an ice bath (~0 °C), was treated with either NBS (13.6 mg, 0.075 mmol) or NIS (17.1 mg, 0.076 µmol), in one portion. The cold bath was removed, and stirring was maintained for 16 h [24]. The reaction mixture was concentrated under a gentle stream of nitrogen and subjected to purification by reversed-phase HPLC; see purification details below.

### 3.3. Boc Deprotection Procedure

A solution of CH_2_Cl_2_/TFA (1:1, 500 μL:500 μL) was added to the Boc-protected thiaplakortone derivative (3–10 mg), and the mixture was stirred at room temperature for 4 h. The products were dried under nitrogen to give the Boc deprotected analogue as the TFA salt [10].

### 3.4. Diversinate™ Synthetic Procedure

Boc-protected thiaplakortone A (**1**, 29.8 mg, 0.076 mmol) was dissolved in CH_2_Cl_2_/H_2_O (2.5:1, 300 μL:120 μL) before the addition of zinc trifluoromethanesulfinate (151.0 mg, 0.460 mmol, 6 equiv.) at room temperature. The mixture was cooled to 4 °C before TBHP (31 μL, 0.460 mmol, 6 equiv.) was slowly added, after which time the stirred mixture was slowly warmed to room temperature over 16 h [27]. The reaction mixture was subsequently subjected to purification by reversed-phase C_18_ HPLC; see purification details below.

### 3.5. HPLC Purification of Reaction Products

Reaction products were pre-adsorbed to C_18_-bonded silica overnight (~1 g) with the dry material packed into a stainless-steel guard cartridge, which was subsequently attached to a semi-preparative reversed-phase HPLC column. Solvent conditions involved a linear gradient from 10% MeOH (0.1% TFA)/90% H_2_O (0.1% TFA) to 100% MeOH (0.1% TFA) over 50 min, which was held for another 10 min at 100% MeOH (0.1% TFA). HPLC flow rates were 4 and 9 mL/min for the Luna and Betasil C_18_ HPLC columns, respectively. Sixty fractions (60 × 1 min) were collected from the start of the HPLC run. Fractions containing UV-active material from each HPLC run were analysed by ^1^H NMR spectroscopy, and relevant fractions with purity >95% were combined to afford the desired products. Compounds **5**, **6**, **9**–**11** were all purified and characterised as their TFA salts.

### 3.6. Experimental Data for Compounds ***3**–**11***

Compound **3**. Orange amorphous powder (2.2 mg, 9%); UV (MeOH) λ_max_ (log ε) 265 (4.85), 394 (4.75) nm; IR (UATR) *ν*_max_ 1747, 1709, 1277, 1199, 1171, 1078, 1025, 975, 712 cm^−1^; ^1^H NMR (800 MHz, DMSO-*d*_6_) *δ*_H_ 1.36 (9H, s, H-15), 2.80 (2H, t, *J* = 7.1 Hz, H-10), 3.16 (2H, dt, *J* = 5.7, 7.1 Hz, H-11), 6.85 (1H, t, *J* = 5.7 Hz, H-12), 7.27 (1H, br s, H-7), 7.39 (1H, s, H-3), 12.97 (1H, br s, H-6); ^13^C NMR (200 MHz, DMSO-*d*_6_) *δ*_C_ 25.7 (C-10), 28.3 (C-15), 39.8 (C-11), 77.6 (C-14), 105.2 (C-2), 111.9 (C-9a), 123.4 (C-8a), 124.7 (C-8), 128.0 (C-5a), 128.9 (C-7), 132.6 (C-3), 140.5 (C-4a), 155.6 (C-13), 167.6 (C-5), 178.4 (C-9); LRESIMS *m*/*z* 470 (100) [^79^Br: M − H]^−^, 472 (100) [^81^Br: M − H]^−^; HRESIMS *m*/*z* 470.0023 [M − H]^−^ (calcd for C_17_H_17_^79^BrN_3_O_6_S, 470.0027).

Compound **4**. Orange amorphous powder (9.1 mg, 33%); UV (MeOH) λ_max_ (log ε) 265 (4.86), 402 (4.69) nm; IR (UATR) *ν*_max_ 3476, 1747, 1704, 1279, 1199, 1161, 1079, 975, 712 cm^−1^; ^1^H NMR (800 MHz, DMSO-*d*_6_) *δ*_H_ 1.35 (9H, s, H-15), 2.74 (2H, t, *J* = 6.9 Hz, H-10), 3.08 (2H, dt, *J* = 5.9, 6.9 Hz, H-11), 6.80 (1H, t, *J* = 5.9 Hz, H-12), 7.41 (1H, s, H-3), 11.38 (1H, br s, H-4), 13.91 (1H, br s, H-6); ^13^C NMR (200 MHz, DMSO-*d*_6_) *δ*_C_ 25.4 (C-10), 28.3 (C-15), 39.4 (C-11), 77.5 (C-14), 105.4 (C-2), 111.4 (C-9a), 116.9 (C-7), 123.5 (C-8a), 123.9 (C-8), 129.0 (C-5a), 132.0 (C-3), 139.8 (C-4a), 155.5 (C-13), 166.2 (C-5), 177.3 (C-9); LRESIMS *m*/*z* 548 (50) [^79^Br_2_: M − H]^−^, 550 (100) [^81^Br^79^Br: M − H]^−^, 552 (50) [^81^Br_2_: M − H]^−^; LRESIMS *m*/*z* 572 (50) [^79^Br_2_: M + Na]^+^, 574 (100) [^81^Br^79^Br: M + Na]^+^, 576 (50) [^81^Br_2_: M + Na]^+^; HRESIMS *m*/*z* 571.9113 [M + Na]^+^ (calcd for C_17_H_17_^79^Br_2_N_3_O_6_SNa, 571.9097).

Compound **5** (TFA Salt). Orange amorphous powder (3.7 mg, 20%); UV (MeOH) λ_max_ (log ε) 262 (5.09), 391 (4.97) nm; IR (UATR) *ν*_max_ 1747, 1709, 1274, 1199, 1164, 972, 714 cm^−1^; ^1^H NMR (800 MHz, DMSO-*d*_6_) *δ*_H_ 2.97 (2H, m, H-10), 3.06 (2H, m, H-11), 7.40 (1H, d, *J* = 2.8 Hz, H-7), 7.43 (1H, br s, H-3), 7.82 (3H, br s, H-12), 11.41 (1H, br s, H-4), 13.15 (1H, br s, H-6); ^13^C NMR (200 MHz, DMSO-*d*_6_) *δ*_C_ 23.5 (C-10), 38.5 (C-11), 105.4 (C-2), 111.7 (C-9a), 121.8 (C-8), 123.4 (C-8a), 128.2 (C-5a), 129.3 (C-7), 132.1 (C-3), 140.0 (C-4a), 167.5 (C-5), 178.4 (C-9); LRESIMS *m*/*z* 372 (100) [^79^Br: M + H]^+^, 374 (100) [^81^Br: M + H]^+^; HRESIMS *m*/*z* 371.9631 [M + H]^+^ (calcd for C_12_H_11_^79^BrN_3_O_4_S, 371.9648).

Compound **6** (TFA Salt). Orange brown gum (2.9 mg, 13%); UV (MeOH) λ_max_ (log ε) 238 (4.85), 399 (4.62) nm; IR (UATR) *ν*_max_ 1747, 1710, 1277, 1199, 1162, 1078, 1026, 975, 712 cm^−1^; ^1^H NMR (800 MHz, DMSO-*d*_6_) *δ*_H_ 2.93 (2H, m, H-10), 2.94 (2H, m, H-11), 7.44 (1H, br s, H-3), 7.85 (3H, br s, H-12), 11.48 (1H, br s, H-4), 14.08 (1H, br s, H-6); ^13^C NMR (200 MHz, DMSO-*d*_6_) *δ*_C_ 23.6 (C-10), 38.9 (C-11), 105.9 (C-2), 111.7 (C-9a), 116.6 (C-7), 121.2 (C-8), 124.5 (C-8a), 129.6 (C-5a), 132.8 (C-3), 140.5 (C-4a), 167.0 (C-5), 178.4 (C-9); LRESIMS *m*/*z* 448 (50) [^79^Br_2_: M − H]^−^, 450 (100) [^81^Br^79^Br: M − H]^−^, 452 (50) [^81^Br_2_: M − H]^−^; HRESIMS *m*/*z* 447.8600 [M − H]^−^ (calcd for C_12_H_8_^79^Br_2_N_3_O_4_S, 447.8608).

Compound **7**. Orange brown gum (1.4 mg, 8%); UV (MeOH) λ_max_ (log ε) 256 (4.55), 404 (4.31) nm; IR (UATR) *ν*_max_ 3458, 1747, 1709, 1278, 1199, 1164, 1078, 1025, 974, 712 cm^−1^; ^1^H NMR (800 MHz, DMSO-*d*_6_) *δ*_H_ 1.35 (9H, s, H-15), 2.79 (2H, t, *J* = 7.1 Hz, H-10), 3.15 (2H, dt, *J* = 5.7, 7.1 Hz, H-11), 6.83 (1H, t, *J* = 5.7 Hz, H-12), 7.25 (1H, br s, H-7), 7.32 (1H, s, H-3); ^13^C NMR (200 MHz, DMSO-*d*_6_) *δ*_C_ 25.7 (C-10), 28.3 (C-15), 39.2 (C-11), 77.6 (C-14), 81.1 (C-2), 109.2 (C-9a), 123.4 (C-8a), 124.6 (C-8), 127.9 (C-5a), 128.9 (C-7), 136.9 (C-3), 140.3 (C-4a), 155.7 (C-13), 167.8 (C-5), 178.5 (C-9); LRESIMS *m*/*z* 518 (100) [M − H]^−^; HRESIMS *m*/*z* 517.9883 [M − H]^−^ (calcd for C_17_H_17_IN_3_O_6_S, 517.9888).

Compound **8**. Orange amorphous powder (2.2 mg, 7%); UV (MeOH) λ_max_ (log ε) 242 (5.24), 413 (4.96) nm; IR (UATR) *ν*_max_ 3413, 2950, 1747, 1709, 1277, 1199, 1163, 1078, 1025, 975, 712 cm^−1^; ^1^H NMR (800 MHz, DMSO-*d*_6_) *δ*_H_ 1.35 (9H, s, H-15), 2.71 (2H, t, *J* = 7.0 Hz, H-10), 3.05 (2H, dt, *J* = 5.9, 7.0 Hz, H-11), 6.75 (1H, t, *J* = 5.9 Hz, H-12), 7.31 (1H, d, *J* = 5.0 Hz, H-3), 11.17 (1H, d, *J* = 5.0 Hz, H-4), 13.59 (1H, s, H-6); ^13^C NMR (200 MHz, DMSO-*d*_6_) *δ*_C_ 27.3 (C-10), 28.4 (C-15), 39.2 (C-11), 77.5 (C-14), 81.0 (C-2), 90.6 (C-7), 108.5 (C-9a), 123.3 (C-8a), 128.9 (C-8), 131.5 (C-5a), 136.3 (C-3), 139.6 (C-4a), 155.5 (C-13), 166.2 (C-5), 177.5 (C-9); LRESIMS *m*/*z* 644 (100) [M − H]^−^; HRESIMS *m*/*z* 643.9873 [M − H]^−^ (calcd for C_17_H_16_I_2_N_3_O_6_S, 643.9878).

Compound **9** (TFA Salt). Orange amorphous powder (2.2 mg, 8%); UV (MeOH) λ_max_ (log ε) 266 (4.56), 398 (4.46) nm; IR (UATR) *ν*_max_ 1747, 1709, 1277, 1199, 1164, 1078, 1054, 1025, 975, 712 cm^−1^; ^1^H NMR (800 MHz, DMSO-*d*_6_) *δ*_H_ 2.97 (2H, m, H-10), 3.05 (2H, m, H-11), 7.32 (1H, d, *J* = 5.9 Hz, H-3), 7.38 (1H, d, *J* = 2.9 Hz, H-7), 7.77 (3H, br s, H-12), 11.23 (1H, br d, *J* = 5.9 Hz, H-4), 13.11 (1H, br d, *J* = 2.9 Hz, H-6); ^13^C NMR (200 MHz, DMSO-*d*_6_) *δ*_C_ 23.5 (C-10), 38.6 (C-11), 81.0 (C-2), 109.0 (C-9a), 121.7 (C-8), 123.4 (C-8a), 128.2 (C-5a), 129.4 (C-7), 136.3 (C-3), 139.9 (C-4a), 167.7 (C-5), 178.6 (C-9); LRESIMS *m*/*z* 418 (100) [M − H]^−^; HRESIMS *m*/*z* 417.9355 [M − H]^−^ (calcd for C_12_H_9_IN_3_O_4_S, 417.9364).

Compound **10** (TFA Salt). Orange amorphous powder (1.4 mg, 4%); UV (MeOH) λ_max_ (log ε) 240 (4.84), 413 (4.68) nm; IR (UATR) *ν*_max_ 3454, 2963, 1751, 1713, 1281, 1200, 1160, 1079, 1027, 973, 714 cm^−1^; ^1^H NMR (800 MHz, DMSO-*d*_6_) *δ*_H_ 2.90 (2H, m, H-10), 2.90 (2H, m, H-11), 7.32 (1H, d, *J* = 5.9 Hz, H-3), 7.86 (3H, br s, H-12), 11.26 (1H, d, *J* = 5.9 Hz, H-4), 13.74 (1H, br s, H-6); ^13^C NMR (200 MHz, DMSO-*d*_6_) *δ*_C_ 25.1 (C-10), 38.3 (C-11), 81.2 (C-2), 90.3 (C-7), 108.3 (C-9a), 123.4 (C-8a), 125.9 (C-8), 131.8 (C-5a), 136.3 (C-3), 139.8 (C-4a), 166.4 (C-5), 177.7 (C-9); LRESIMS *m*/*z* 544 (100) [M − H]^−^; HRESIMS *m*/*z* 543.8312 [M − H]^−^ (calcd for C_12_H_8_I_2_N_3_O_4_S, 543.8330).

Compound **11** (TFA Salt). Yellow gum (4.8 mg, 15%); UV (MeOH) λ_max_ (log ε) 239 (4.82), 346 (4.35) nm; IR (UATR) *ν*_max_ 1747, 1709, 1277, 1199, 1165, 1078, 1026, 975, 712 cm^−1^; ^1^H NMR (800 MHz, DMSO-*d*_6_) *δ*_H_ 2.94 (2H, m, H-11), 3.09 (2H, m, H-10), 7.64 (1H, br s, H-3), 7.93 (3H, br s, H-12); ^13^C NMR (200 MHz, DMSO-*d*_6_) *δ*_C_ 22.3 (C-10), 38.9 (C-11), 112.1 (q, ^2^*J*_CF_ = 32.5 Hz, C-2), 121.8 (br q, ^3^*J*_CF_ = 2.1 Hz, C-8), 122.2 (q, ^1^*J*_CF_ = 231.2 Hz, C-14), 134.8 (q, ^3^*J*_CF_ = 5.8 Hz, C-3), 124.4 (C-8a), 140.4 (C-4a), 168.9 (C-5), 177.3 (C-9); LRESIMS *m*/*z* 430 (100) [M + H]^+^; HRESIMS *m*/*z* 430.0274 [M + H]^+^ (calcd for C_14_H_10_F_6_N_3_O_4_S, 430.0291). Note: Several ^13^C NMR signals could not be assigned for **11** due to low signal-to-noise in the ^13^C NMR spectrum and extensive splitting of certain signals due to ^19^F couplings and the small amount of material available. Furthermore, ^13^C NMR assignments for C-13, C-7, C-5a and C-9a were not possible following 2D NMR data analysis due to the lack of sharp exchangeable ^1^H NMR signals for H-4 and H-6, which in other related analogues (e.g., compound **10**) had shown HMBC correlations that enabled complete and definitive ^13^C NMR chemical shift assignments.

### 3.7. In Vitro Antimalarial Image-Based Asexual Assay

*Plasmodium falciparum* 3D7 and Dd2 parasites were cultured in RPMI1640 (Life Technologies, Camarillo, CA, USA) supplemented with 2.5 mg/mL Albumax II, 5% AB human serum, 25 mM HEPES and 0.37 mM hypoxanthine. Ring-stage parasites were treated with compounds following two rounds of sorbitol synchronisation as previously described [8]. Artesunate, pyrimethamine and puromycin were incorporated as controls. Following incubation of assay plates for 72 h at 37 °C, and 5% CO_2_ and 5% O_2_, parasites were stained with 2-(4-amidinophenyl)-1*H*-indole-6-carboxamidine (DAPI) and imaged using an Opera QEHS confocal imaging system (PerkinElmer, Waltham, MA, USA). Images were analysed as previously described using Acapella spot detection software (PerkinElmer, Waltham, MA, USA) [8].

### 3.8. In Vitro Cytotoxicity Assay

Human embryonic kidney cells (HEK293) were maintained in DMEM (Life Technologies, Camarillo, CA, USA) containing 10% FBS (Hyclone™ ThermoFisher, Melbourne, Australia). Cytotoxicity testing was undertaken as previously described [9]. In brief, 5 μL of test compound was added/well of black/clear tissue culture treated 384-well plates containing 3000 adherent HEK293 cells/well in 45 μL and incubated 72 h at 37 °C in 5% CO_2_. After incubation, the supernatant was removed and replaced with 40 μL of 10% Alamar Blue per well. Plates were incubated for 5–6 h and measured for fluorescence at 530 nm excitation and 595 nm emission. The % inhibition was calculated using 0.4% DMSO (no inhibition) and 5 μM puromycin (100% inhibition) data. IC_50_ values were obtained by plotting % inhibition against log dose using GraphPad Prism v.6 (San Diego, CA, USA), nonlinear regression with a variable slope plot [9].

## 4. Conclusions

In total, nine new halogenated analogues based on the thiaplakortone A skeleton were synthesised and fully characterised by NMR, UV, IR and MS. Whilst the yields of the late-stage halogenation reactions were not as high as expected, sufficient quantities of all new analogues were obtained in high purity that enabled antimalarial and cytotoxicity profiling. Addition of Br, I or CF_3_ groups around the tricyclic thiaplakortone A core was not advantageous, with antimalarial activity decreasing substantially compared to the potent natural product. Minimal to no cytotoxicity was recorded for the new halogenated derivatives at the top screening concentration of 80 μM. The outcomes from these proof-of-concept late-stage functionalisation studies suggest that with respect to antimalaria potential, further halogenation efforts at C-2 and C-7 are not warranted for this particular scaffold. All new chemistry produced during this project will be added to the Davis Open Access Natural Product-based Library [31,32] and screened in other bioassays in the future. Thus, while compounds **3**–**11** will not directly impact antimalarial drug discovery and development, they may provide future leads for other biomedical applications or be the source of new probes for chemical biology research.

## Figures and Tables

**Table 1:** In silico Physicochemical Parameters for Compounds **1**–**11** ^a^.

Compound	MW	cLog P	cLog D_7.4_	HBD	HBA	Ro5 Violations
**1**	293	−1.52	−3.19	3	6	0
**2**	393	−0.02	−0.02	3	6	0
**3**	471	1.56	1.56	3	6	0
**4**	549	2.02	1.95	3	6	0
**5**	371	0.06	−1.61	3	6	0
**6**	449	0.15	−0.94	3	6	0
**7**	519	1.72	1.72	3	6	1
**8**	645	2.27	2.21	3	6	1
**9**	419	0.22	−1.45	3	6	0
**10**	545	0.50	−0.61	3	6	1
**11**	429	−0.42	−1.50	3	6	0

^a^ In silico calculations performed using ChemAxon’s online prediction software Chemicalize [28]. MW = molecular weight (Da), major isotopic species shown; cLog P = calculated logarithm of the partition coefficient of a compound between *n*-octanol and water; cLog D_7.4_ = calculated logarithm of the partition coefficient of a compound between *n*-octanol and water at a pH of 7.4; HBA = H-bond acceptors; HBD = H-bond donors; Ro5 = Rule of Five [29].

**Table 2:** Biological Data for Compounds **1**–**11**.

**Compound**	***Pf*** **3D7 ^a^** **IC_50_ ± SD µM**	***Pf*** **Dd2 ^b^** **IC_50_ ± SD µM**	**HEK293 ^c^** **IC_50_ ± SD µM**	**SI for 3D7 ^d^**	**SI for Dd2 ^d^**
**1**	0.061 ± 0.009	0.008 ± 0.001	1.32 ± 0.092	22	150
**2**	0.227 ± 0.044	0.086 ± 0.012	5.53 ± 0.021	24	64
**3**	3.515 ± 0.350	0.583 ± 0.094	>80	>23	>137
**4**	1.373 ± 0.117	2.060 ± 0.192	>80	>58	>39
**5**	0.559 ± 0.145	0.058 ± 0.016	>80	>143	>1379
**6**	1.653 ± 0.194	0.487 ± 0.098	>80	>48	>164
**7**	5.623 ± 0.383	1.194 ± 0.270	>80	>14	>67
**8**	1.623 ± 0.154	0.659 ± 0.119	>80	>49	>121
**9**	2.223 ± 0.248	0.317 ± 0.008	>80	>36	>252
**10**	1.685 ± 0.316	0.725 ± 0.244	>80	>47	>110
**11**	6.800 ± 2.444	4.550 ± 1.154	>80	>12	>18
**Controls**	***Pf*** **3D7 ^a^** **IC_50_ ± SD nM**	***Pf*** **Dd2 ^b^** **IC_50_ ± SD nM**	**HEK293 ^c^** **IC_50_ ± SD nM**	**SI for 3D7 ^d^**	**SI for Dd2 ^d^**
Pyrimethamine	3.75 ± 0.30	IA ^e^	>40,000	>10,666	-
Artesunate	0.49 ± 0.20	0.38 ± 0.01	>4000	>8163	>10,526
Puromycin	31.95 ± 16.2	17.00 ± 3.96	1044 ± 66	33	61

^a^ *Pf* 3D7 = *Plasmodium falciparum* (chloroquine-sensitive strain); ^b^ *Pf* Dd2 = *Plasmodium falciparum* (chloroquine, pyrimethamine and mefloquine drug-resistant strain); ^c^ All compounds and controls tested for cytotoxicity against human embryonic kidney cells (HEK293) in order to determine selectivity index (SI); ^d^ SI = HEK293 IC_50_/parasite IC_50_; ^e^ IA = inactive at 40 µM; SD = standard deviation. Note: compounds **1**–**11** and all positive controls were tested in n = 4 biological replicates for the 3D7 and Dd2 malaria assays and n = 2 for the cytotoxicity HEK293 assay. Activity data are presented as mean (±SD).

## Data Availability

The data presented in this study are available in the article.

## Supplementary Materials

The following supporting information can be downloaded at: https://www.mdpi.com/article/10.3390/md21050317/s1, 1D and 2D NMR data for compounds **3**–**11**, synthetic route for thiaplakortone A [10].
