# Supplementary material for: Synthesis and Antimalarial Evaluation of Halogenated Analogues of Thiaplakortone A
Source: Mar Drugs. 2023 May 22;21(5):317. doi: 10.3390/md21050317 (PMC10221708; doi:10.3390/md21050317)
Supplement: Supplementary file 1 [file marinedrugs-21-00317-s001.zip › marinedrugs-2364806-supplementary.pdf]

Synthesis and Antimalarial Evaluation of Halogenated Analogues of Thiaplakortone A

Folake A. Egbewande [1], Brett D. Schwartz [1], Sandra Duffy [1,2], Vicky M. Avery [1,2] and Rohan A. Davis [1,3,*]

[1] Griffith Institute for Drug Discovery, School of Environment and Science, Griffith University, Brisbane, QLD 4111, Australia

[2] Discovery Biology, Centre for Cellular Phenomics, Griffith University, Nathan, QLD 4111, Australia

[3] NatureBank, Griffith University, Nathan, QLD 4111, Australia

*Correspondence: r.davis@griffith.edu.au

Table of Contents

S1: ^{1}H NMR Spectrum for Compound **3** in DMSO-d_6 4
S2: ^{13}C NMR Spectrum for Compound **3** in DMSO-d_6 4
S3: COSY NMR Spectrum for Compound **3** in DMSO-d_6 5
S4: HSQC NMR Spectrum for Compound **3** in DMSO-d_6 5
S5: HMBC NMR Spectrum for Compound **3** in DMSO-d_6 6
S6: ^{1}H NMR Spectrum for Compound **4** in DMSO-d_6 7
S7: ^{13}C NMR Spectrum for Compound **4** in DMSO-d_6 7
S8: COSY NMR Spectrum for Compound **4** in DMSO-d_6 8
S9: HSQC NMR Spectrum for Compound **4** in DMSO-d_6 8
S10: HMBC NMR Spectrum for Compound **4** in DMSO-d_6 9
S11: ^{1}H NMR Spectrum for Compound **5** in DMSO-d_6 10
S12: ^{13}C NMR Spectrum for Compound **5** in DMSO-d_6 10
S13: COSY NMR Spectrum for Compound **5** in DMSO-d_6 11
S14: HSQC NMR Spectrum for Compound **5** in DMSO-d_6 11
S15: HMBC NMR Spectrum for Compound **5** in DMSO-d_6 12
S16: ^{1}H NMR Spectrum for Compound **6** in DMSO-d_6 13
S17: ^{13}C NMR Spectrum for Compound **6** in DMSO-d_6 13
S18: COSY NMR Spectrum for Compound **6** in DMSO-d_6 14
S19: HSQC NMR Spectrum for Compound **6** in DMSO-d_6 14
S20: HMBC NMR Spectrum for Compound **6** in DMSO-d_6 15
S21: ^{1}H NMR Spectrum for Compound **7** in DMSO-d_6 16
S22: ^{13}C NMR Spectrum for Compound **7** in DMSO-d_6 16
S23: COSY NMR Spectrum for Compound **7** in DMSO-d_6 17
S24: HSQC NMR Spectrum for Compound **7** in DMSO-d_6 17
S25: HMBC NMR Spectrum for Compound **7** in DMSO-d_6 18
S26: ^{1}H NMR Spectrum for Compound **8** in DMSO-d_6 19
S27: ^{13}C NMR Spectrum for Compound **8** in DMSO-d_6 19
S28: COSY NMR Spectrum for Compound **8** in DMSO-d_6 20
S29: HSQC NMR Spectrum for Compound **8** in DMSO-d_6 20
S30: HMBC NMR Spectrum for Compound **8** in DMSO-d_6 21
S31: ^{1}H NMR Spectrum for Compound **9** in DMSO-d_6 22

S32: ^{13}C NMR Spectrum for Compound **9** in DMSO-d_6 22
S33: COSY NMR Spectrum for Compound **9** in DMSO-d_6 23
S34: HSQC NMR Spectrum for Compound **9** in DMSO-d_6 23
S35: HMBC NMR Spectrum for Compound **9** in DMSO-d_6 24
S36: ^{1}H NMR Spectrum for Compound **10** in DMSO-d_6 25
S37: ^{13}C NMR Spectrum for Compound **10** in DMSO-d_6 25
S38: COSY NMR Spectrum for Compound **10** in DMSO-d_6 26
S39: HSQC NMR Spectrum for Compound **10** in DMSO-d_6 26
S40: HMBC NMR Spectrum for Compound **10** in DMSO-d_6 27
S41: ^{1}H NMR Spectrum for Compound **11** in DMSO-d_6 28
S42: ^{13}C NMR Spectrum for Compound **11** in DMSO-d_6 28
S43: COSY NMR Spectrum for Compound **11** in DMSO-d_6 29
S44: HSQC NMR Spectrum for Compound **11** in DMSO-d_6 29
S45: HMBC NMR Spectrum for Compound **11** in DMSO-d_6 30
S46: Synthetic Route for Thiaplakortone A (HCl Salt) 31

S1: ^{1}H NMR Spectrum for Compound **3** in DMSO-d_6

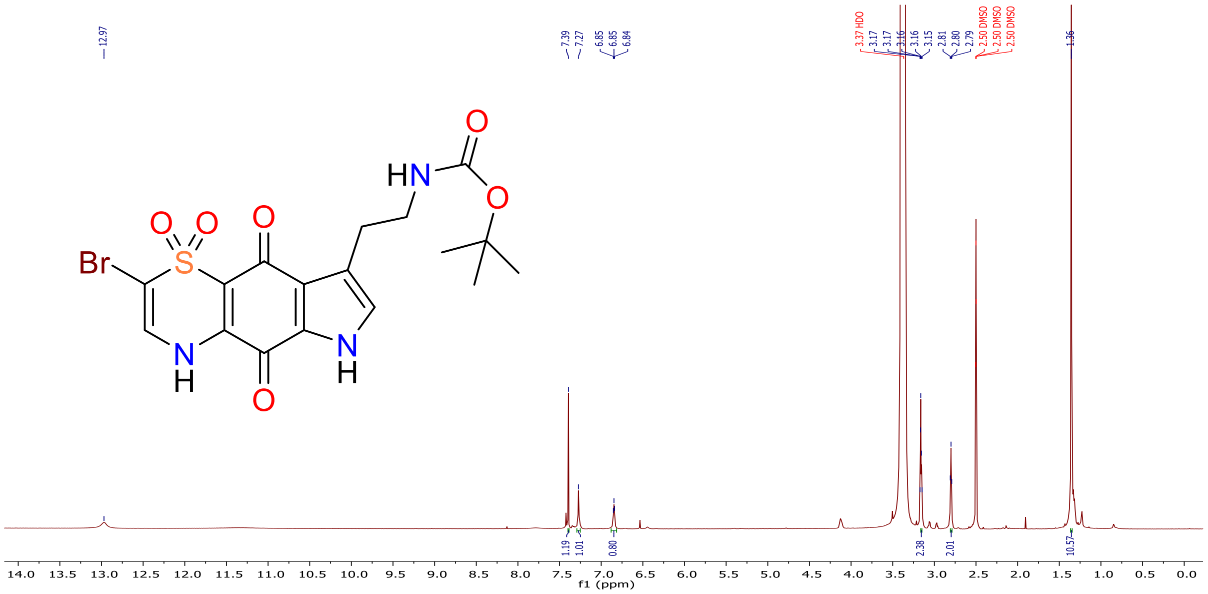

S2: ^{13}C NMR Spectrum for Compound **3** in DMSO-d_6

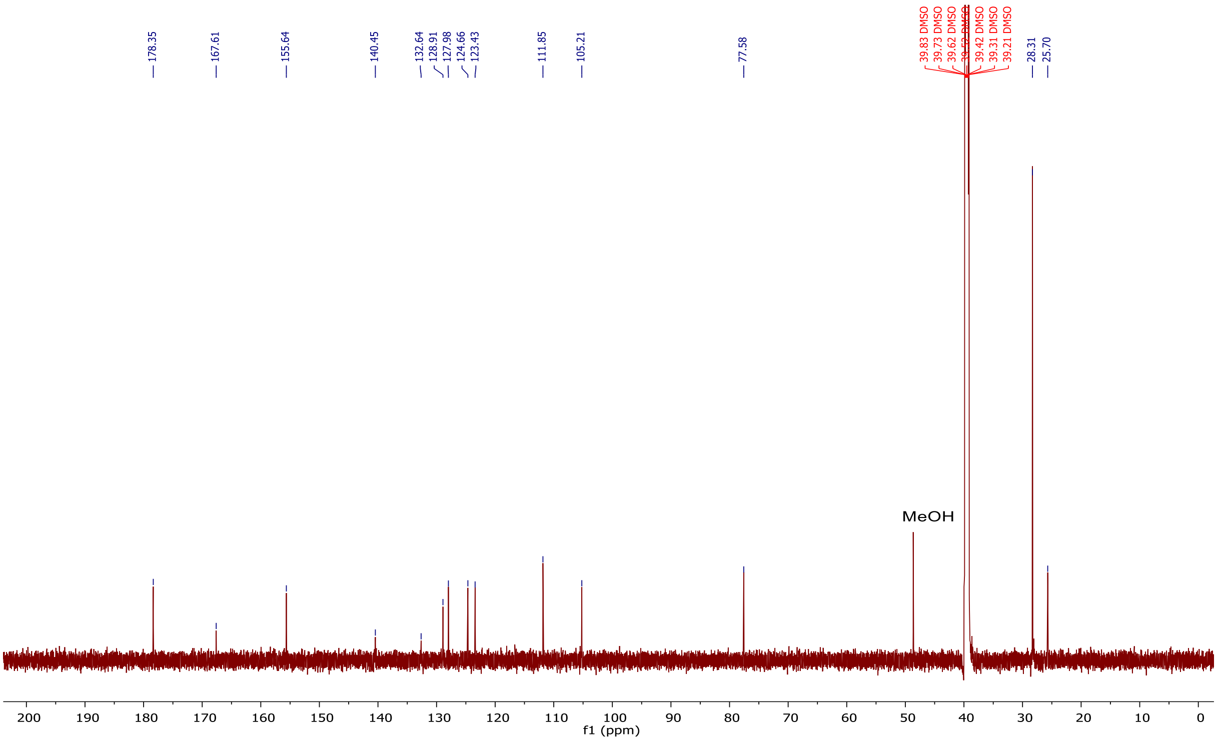

S3: COSY NMR Spectrum for Compound **3** in DMSO-*d*6

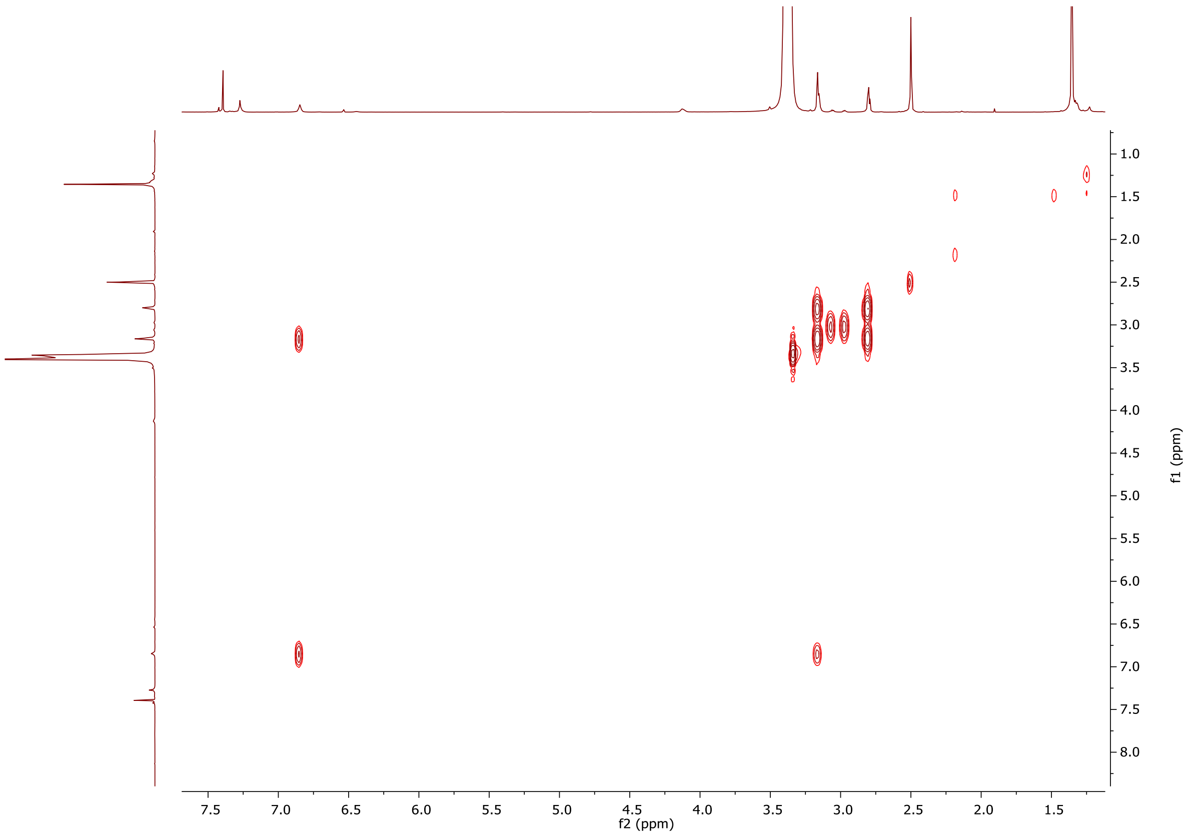

S4: HSQC NMR Spectrum for Compound **3** in DMSO-*d*6

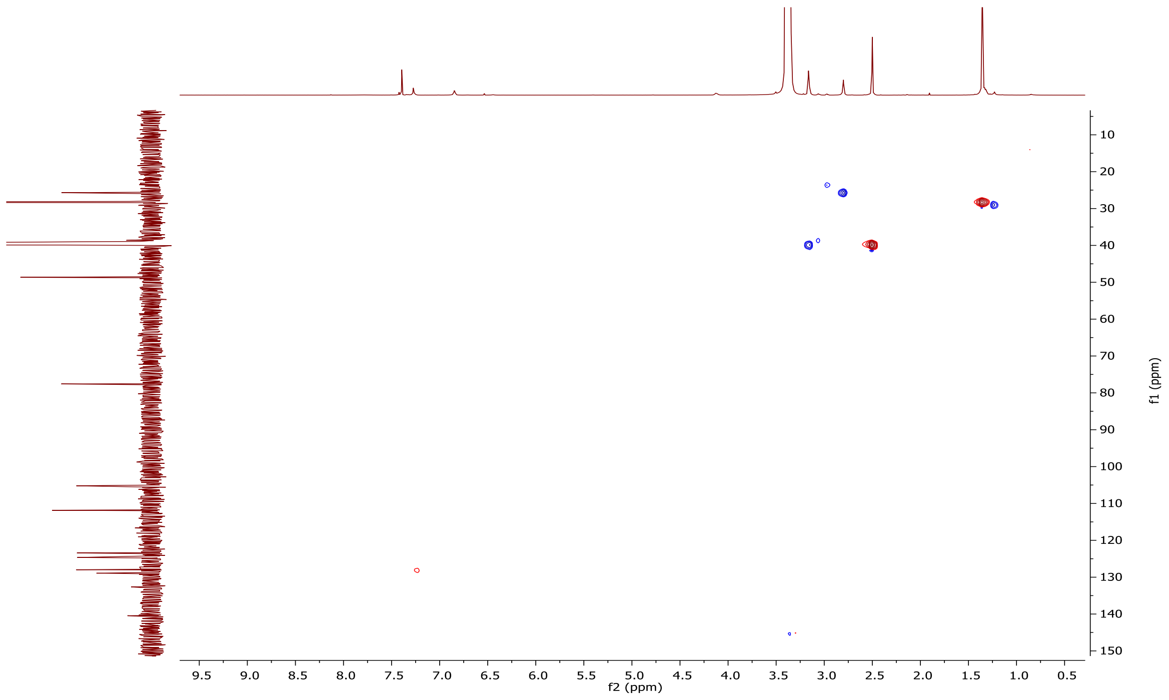

S5: HMBC NMR Spectrum for Compound **3** in DMSO-d_6

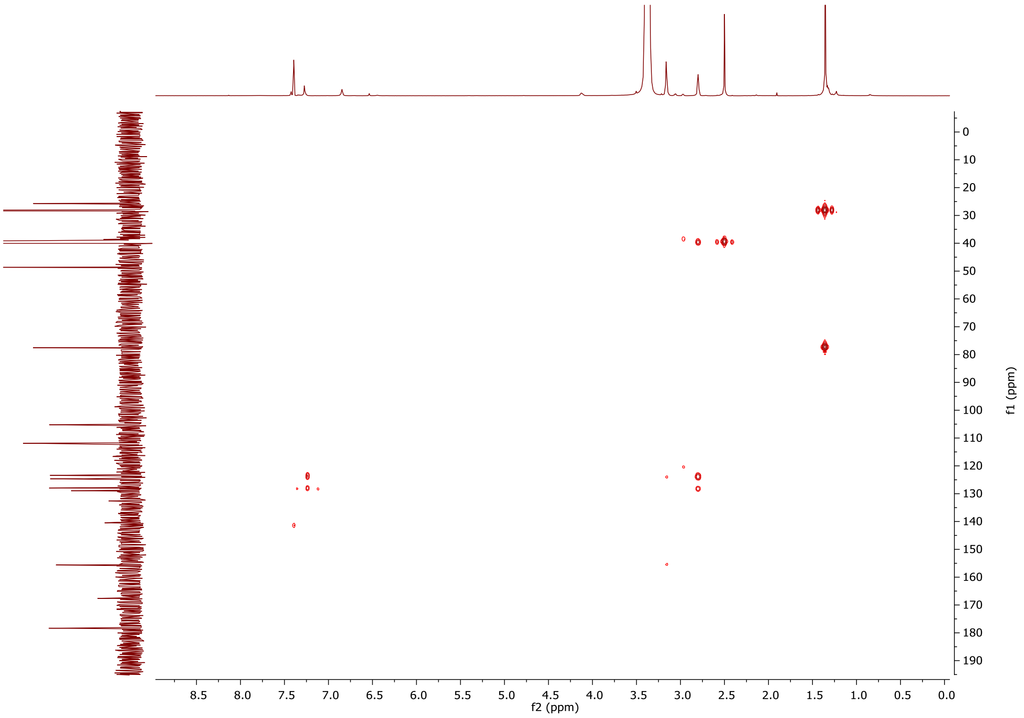

S6: ^{1}H NMR Spectrum for Compound **4** in DMSO-d_6

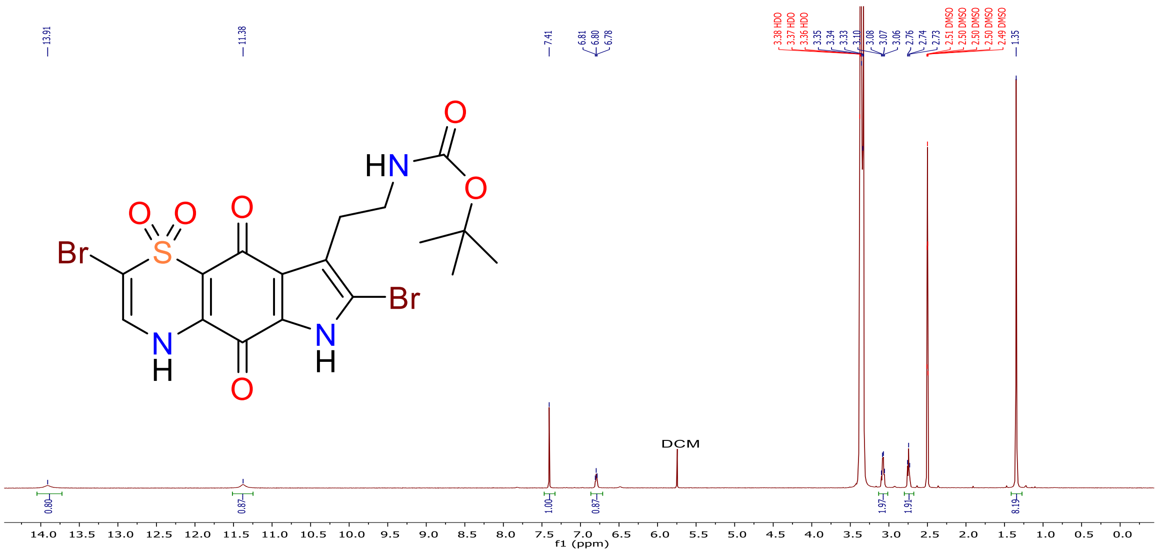

S7: ^{13}C NMR Spectrum for Compound **4** in DMSO-d_6

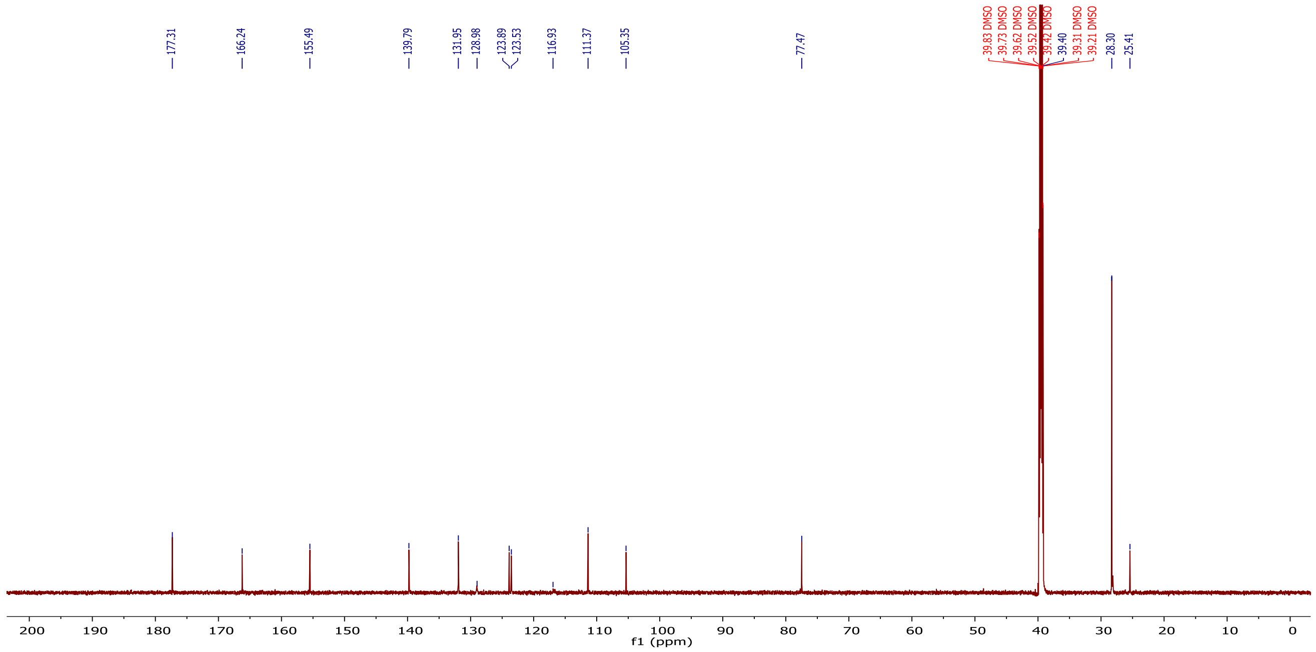

S8: COSY NMR Spectrum for Compound **4** in DMSO-d_6

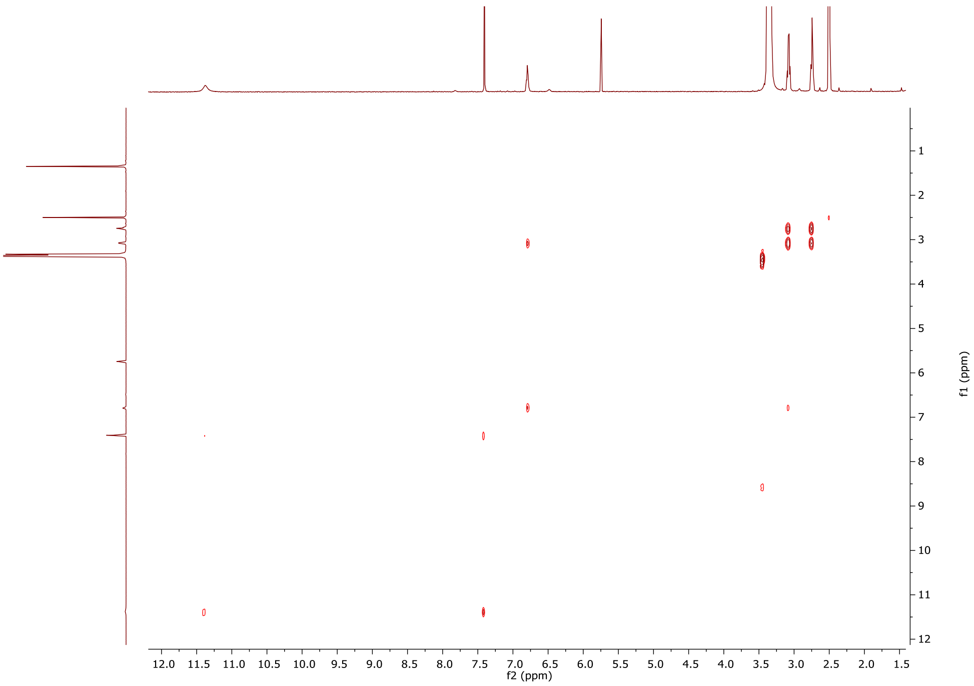

S9: HSQC NMR Spectrum for Compound **4** in DMSO-d_6

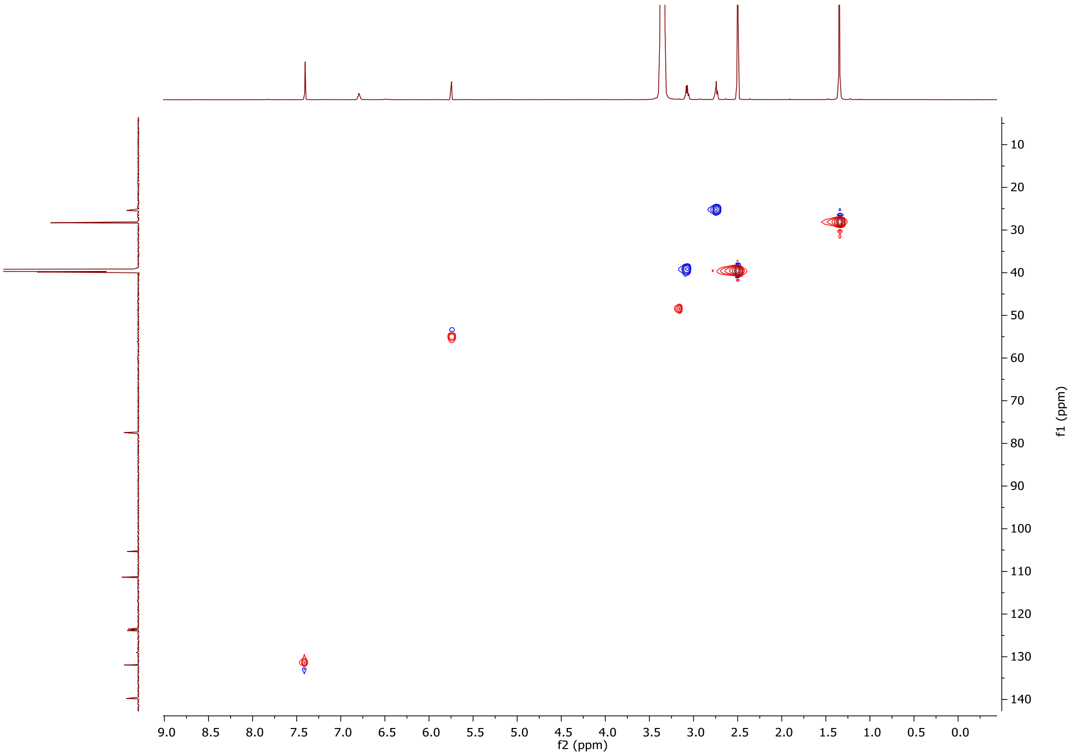

S10: HMBC NMR Spectrum for Compound **4** in DMSO-d_6

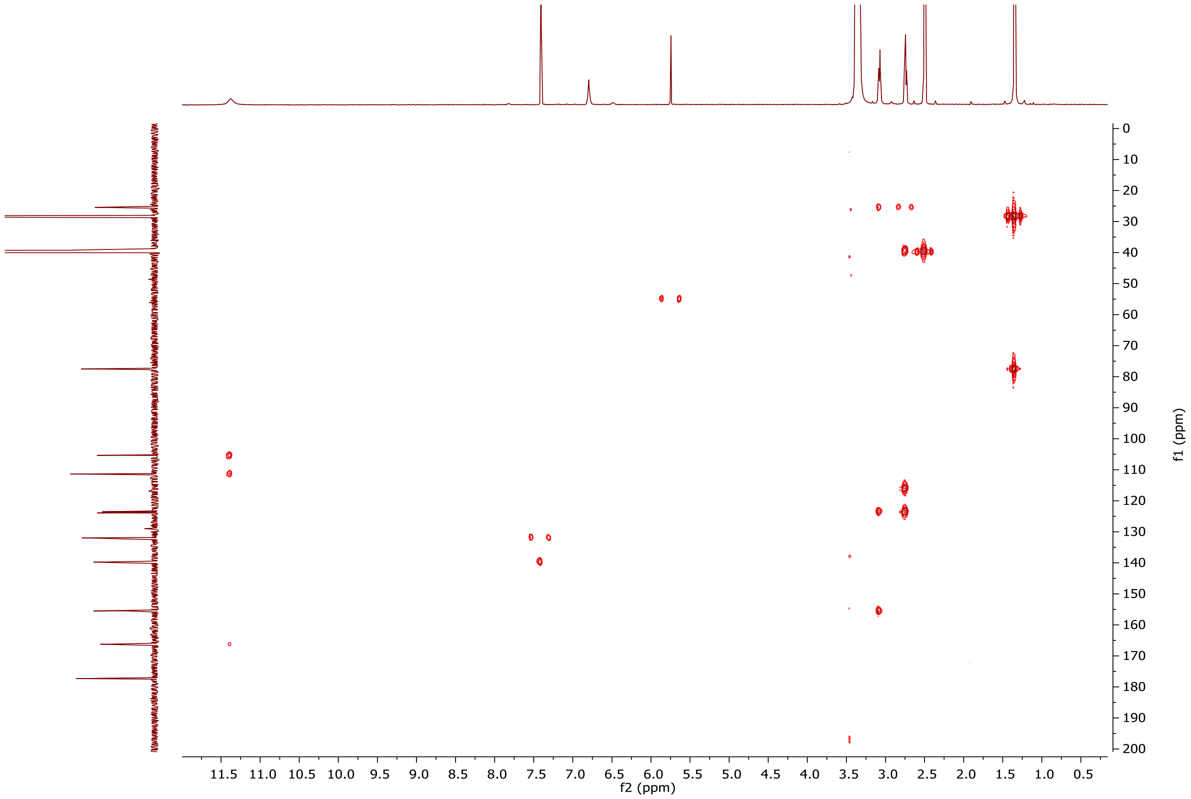

S11: ^{1}H NMR Spectrum for Compound **5** in DMSO-d_6

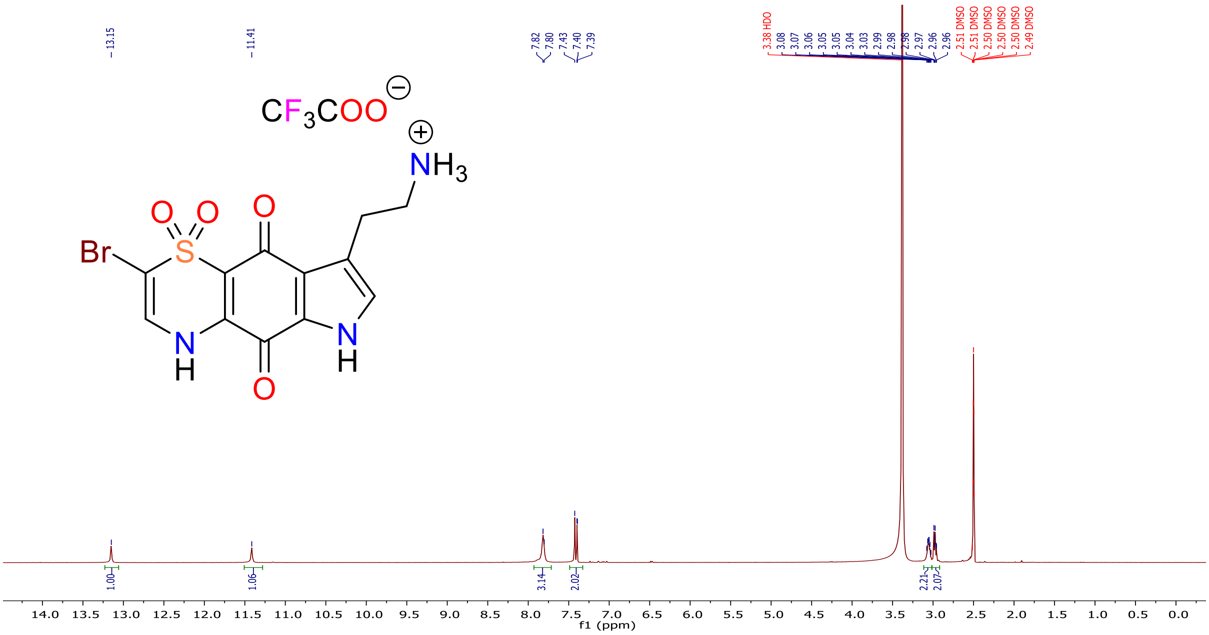

S12: ^{13}C NMR Spectrum for Compound **5** in DMSO-d_6

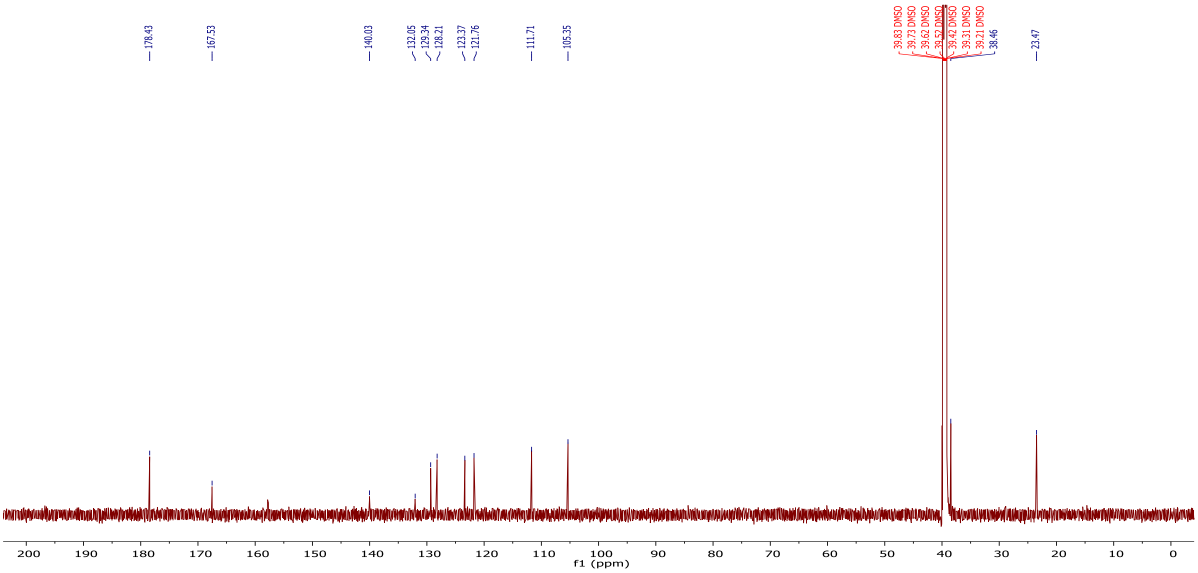

S13: COSY NMR Spectrum for Compound **5** in DMSO-d_6

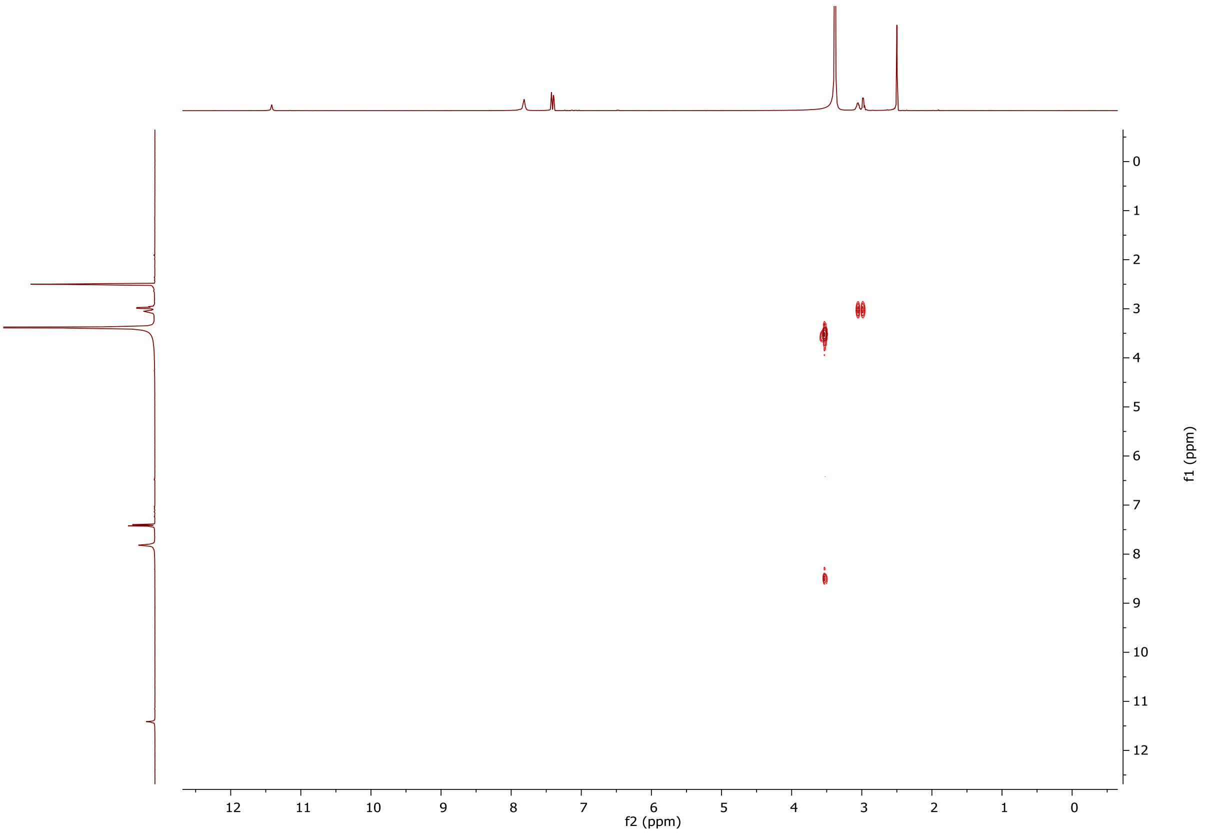

S14: HSQC NMR Spectrum for Compound **5** in DMSO-d_6

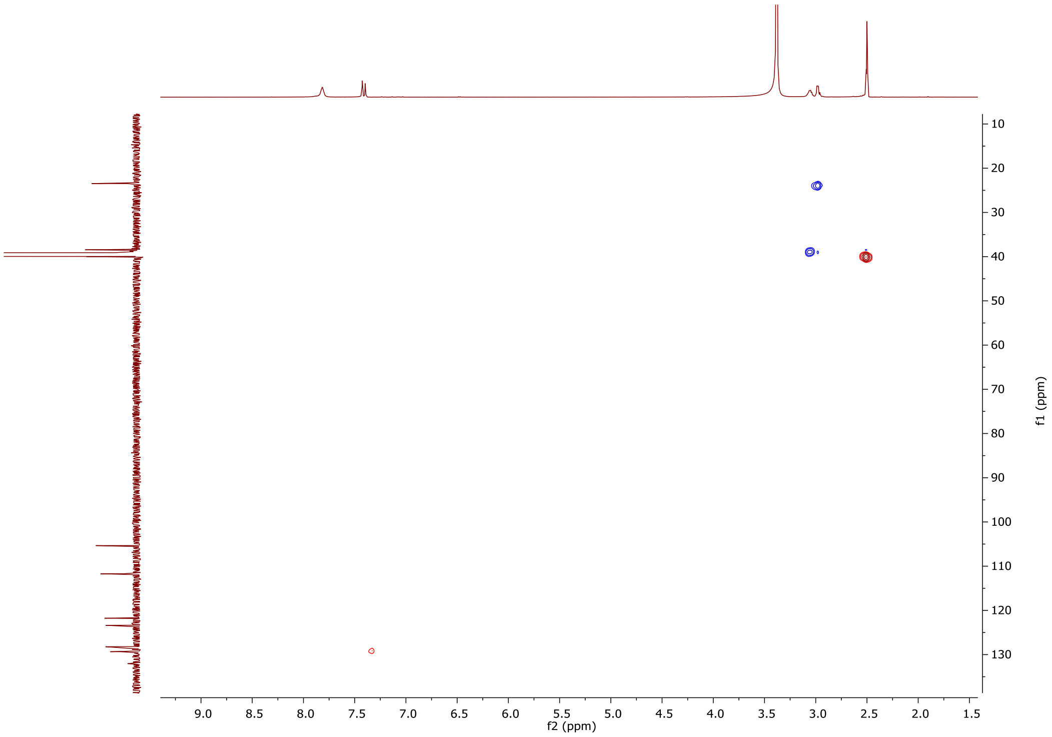

S15: HMBC NMR Spectrum for Compound **5** in DMSO-*d*6

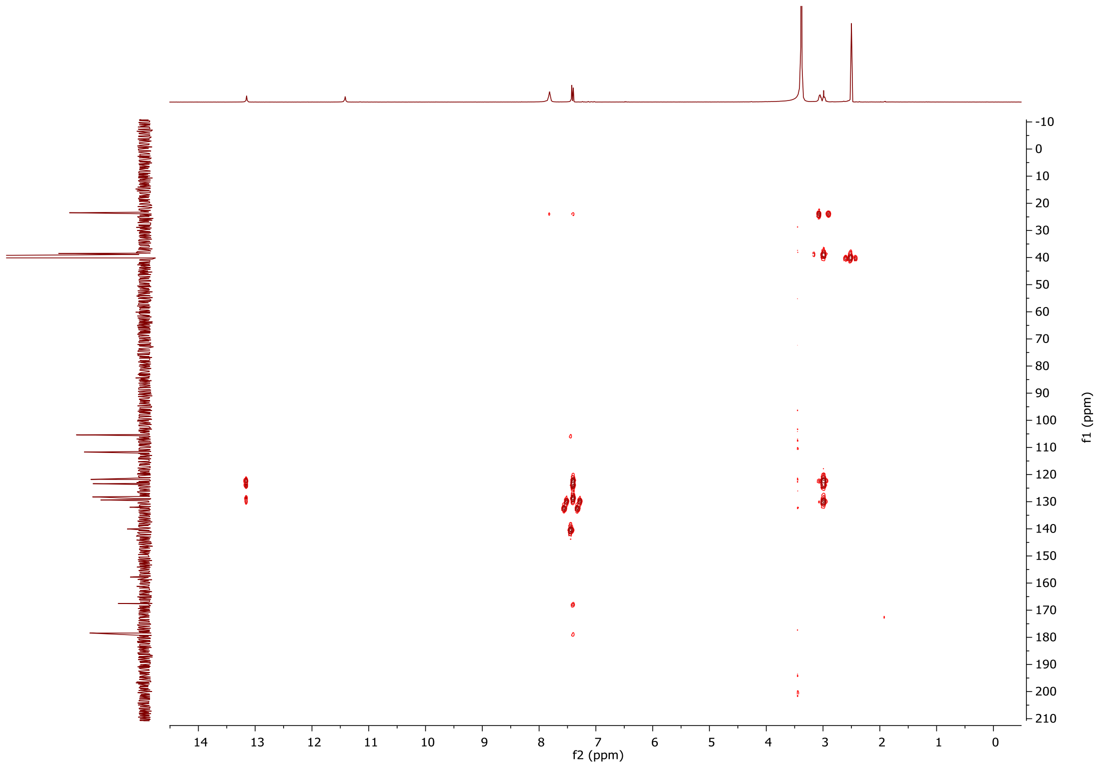

S16: ^{1}H NMR Spectrum for Compound **6** in DMSO-d_6

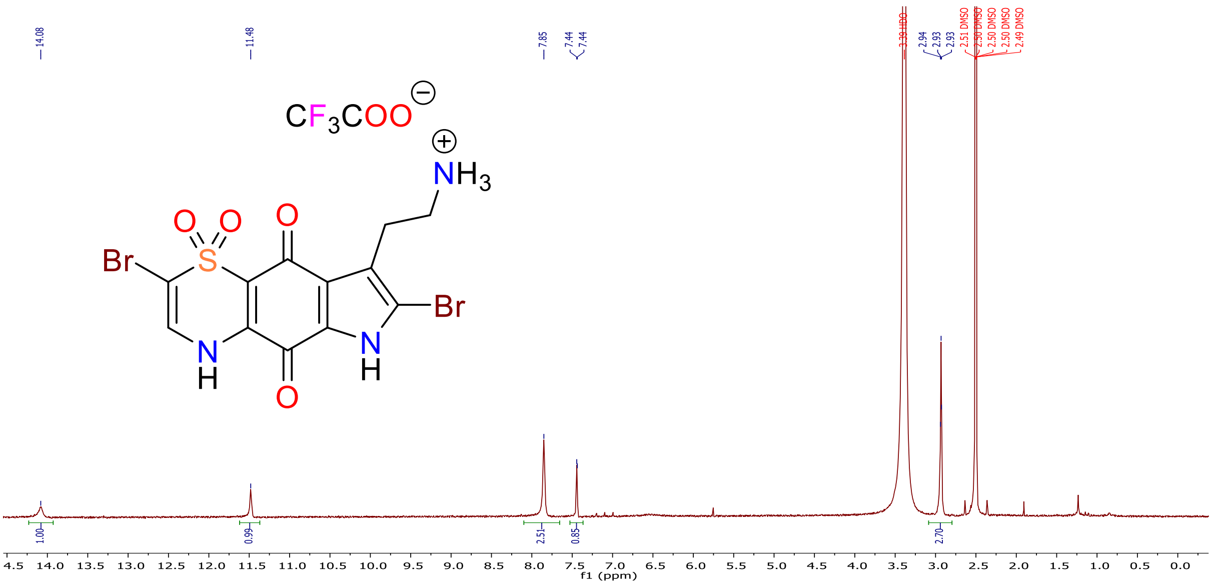

S17: ^{13}C NMR Spectrum for Compound **6** in DMSO-d_6

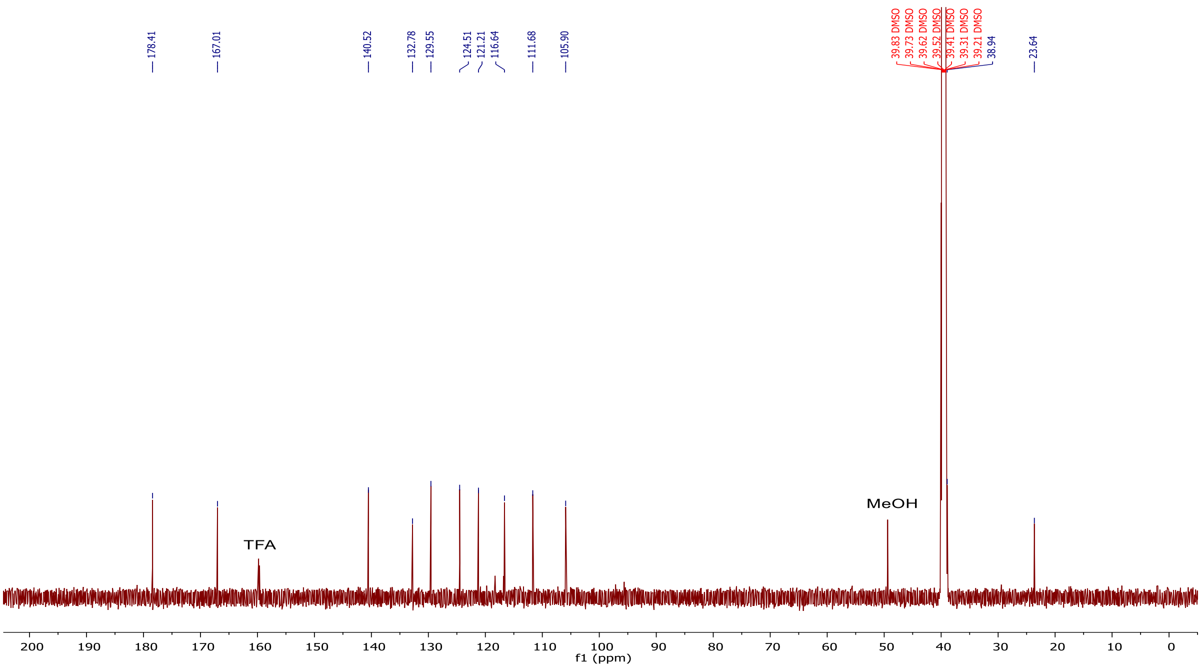

S18: COSY NMR Spectrum for Compound **6** in DMSO-d_6

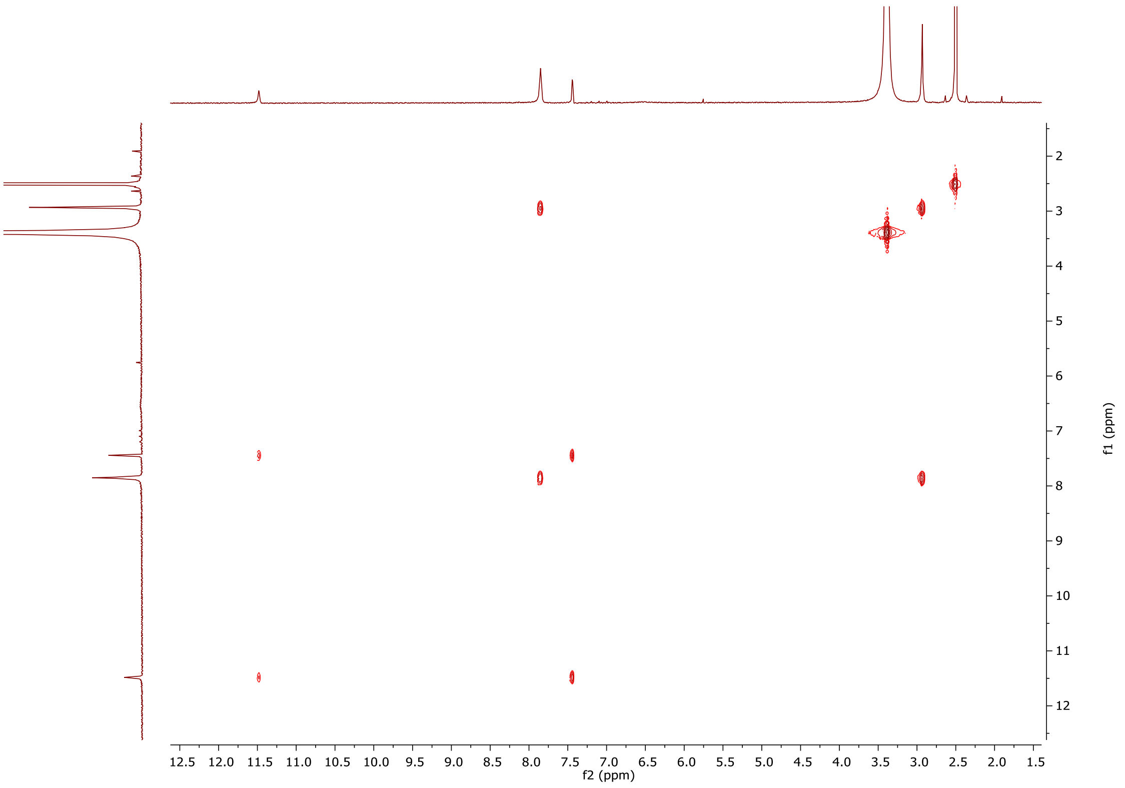

S19: HSQC NMR Spectrum for Compound **6** in DMSO-d_6

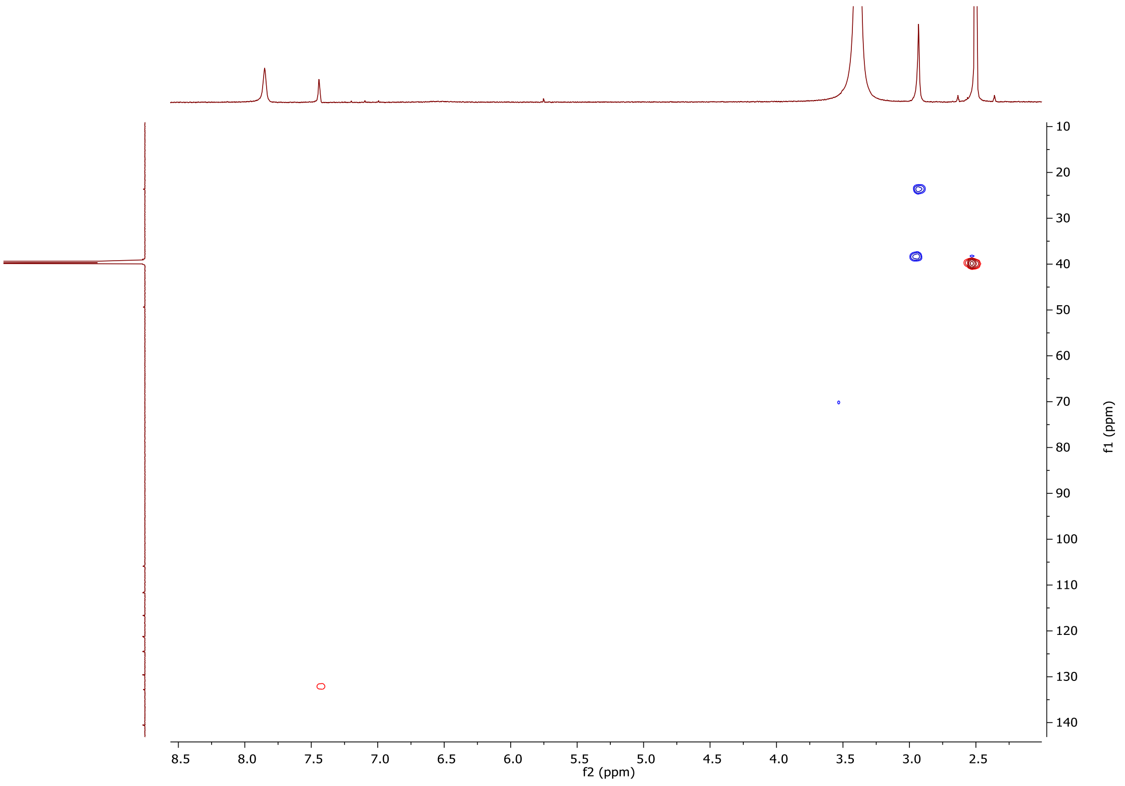

S20: HMBC NMR Spectrum for Compound **6** in DMSO-d_6

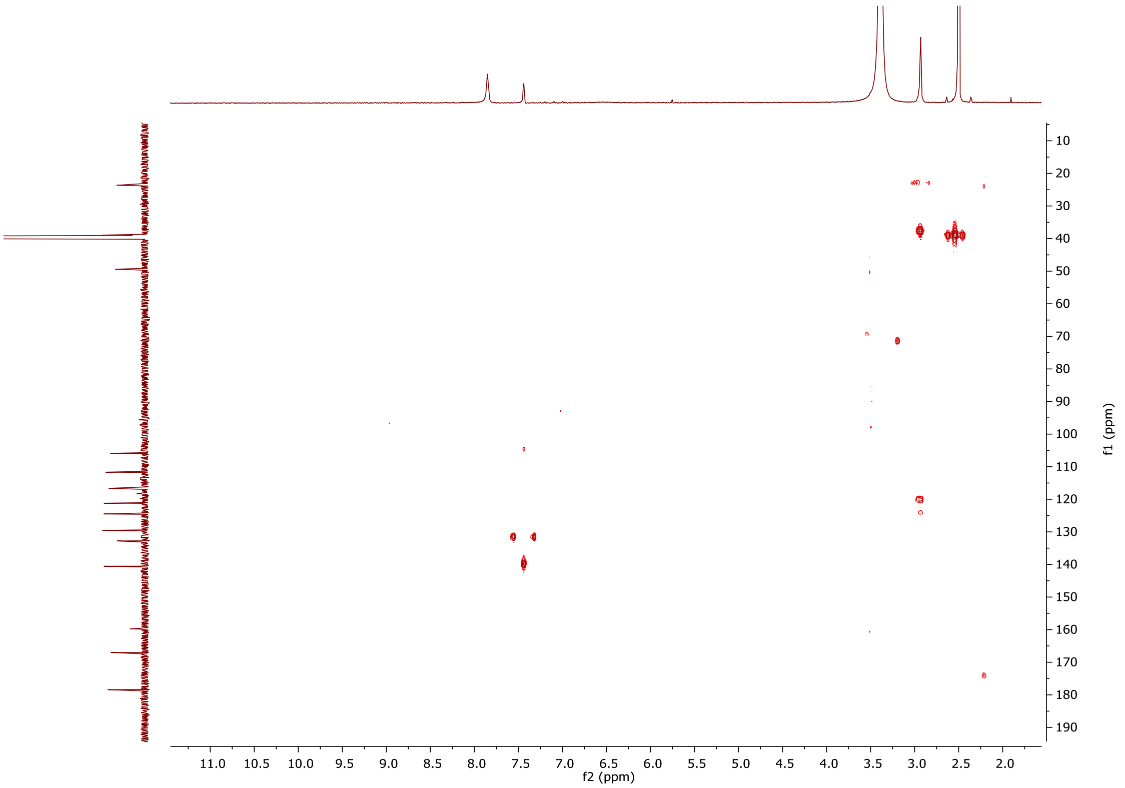

S21: ^{1}H NMR Spectrum for Compound **7** in DMSO-d_6

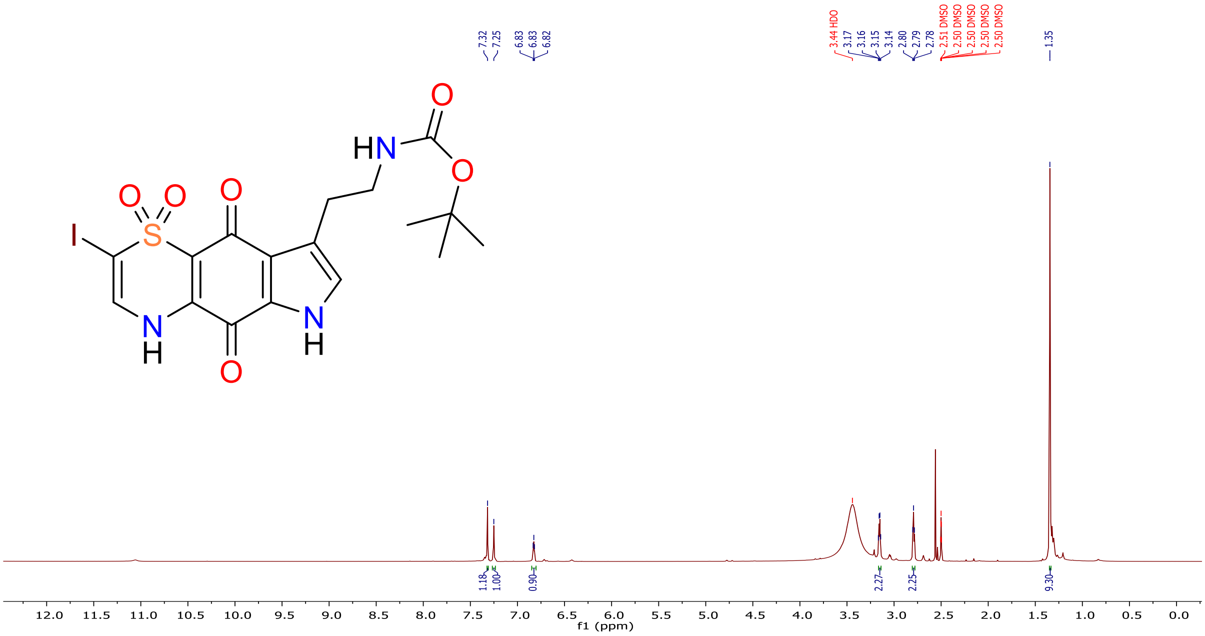

S22: ^{13}C NMR Spectrum for Compound **7** in DMSO-d_6

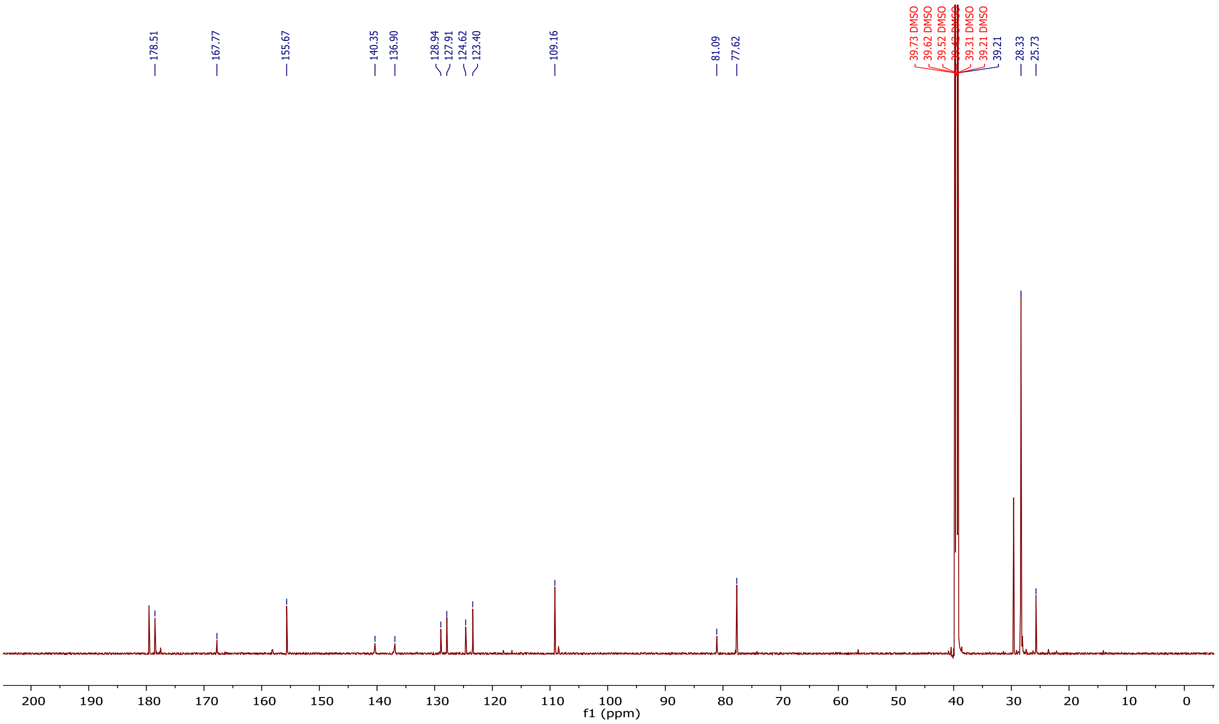

S23: COSY NMR Spectrum for Compound **7** in DMSO-d_6

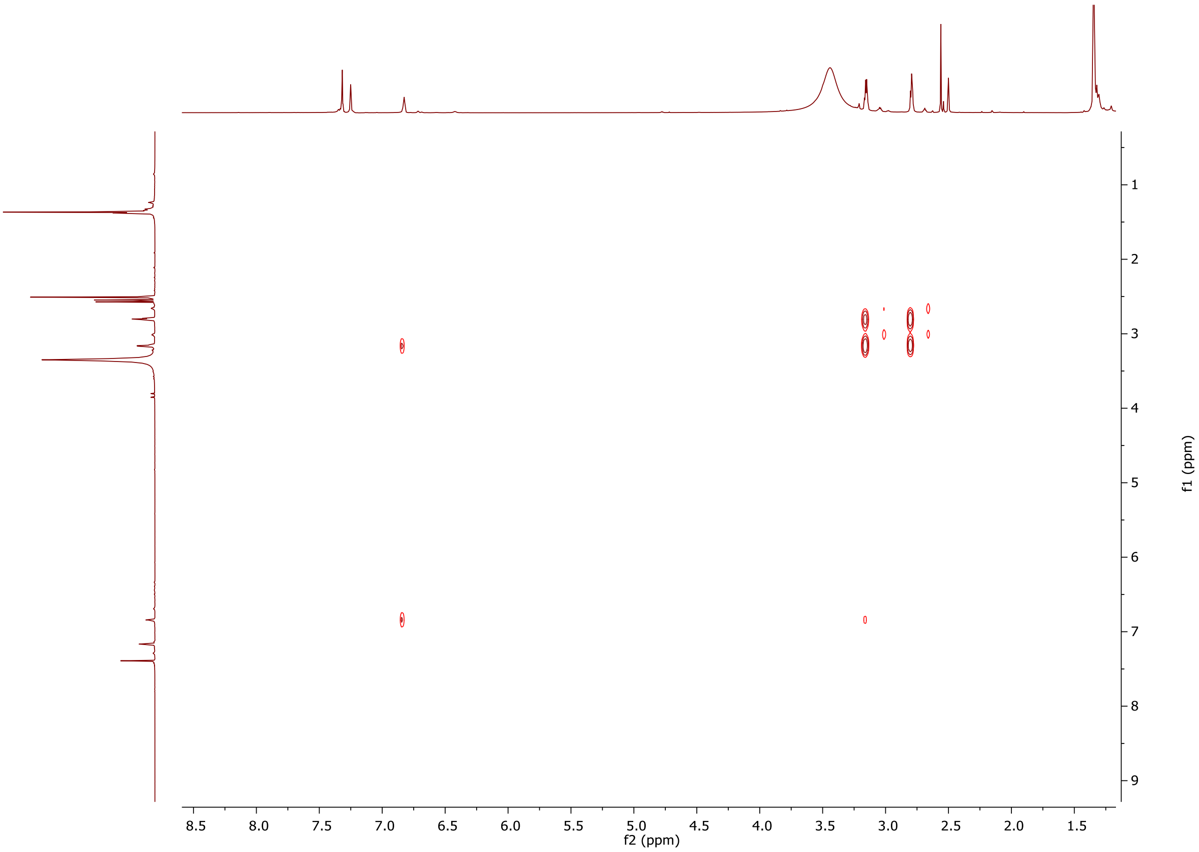

S24: HSQC NMR Spectrum for Compound **7** in DMSO-d_6

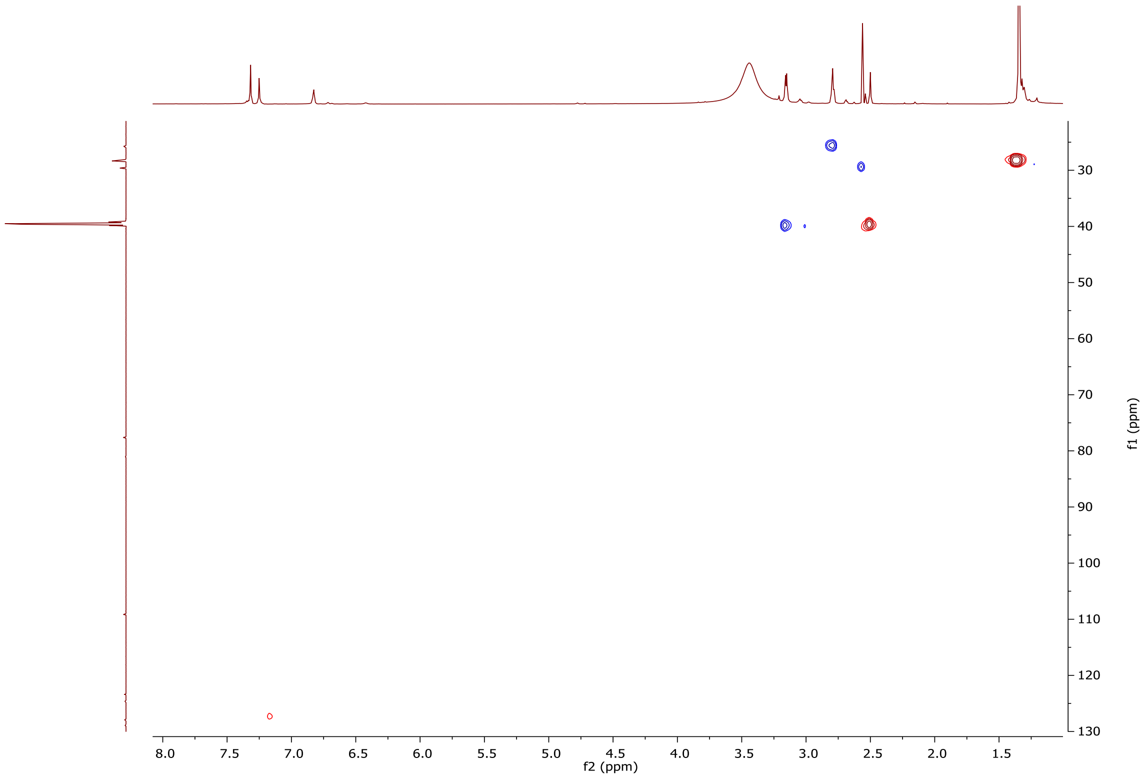

S25: HMBC NMR Spectrum for Compound **7** in DMSO-*d*₆

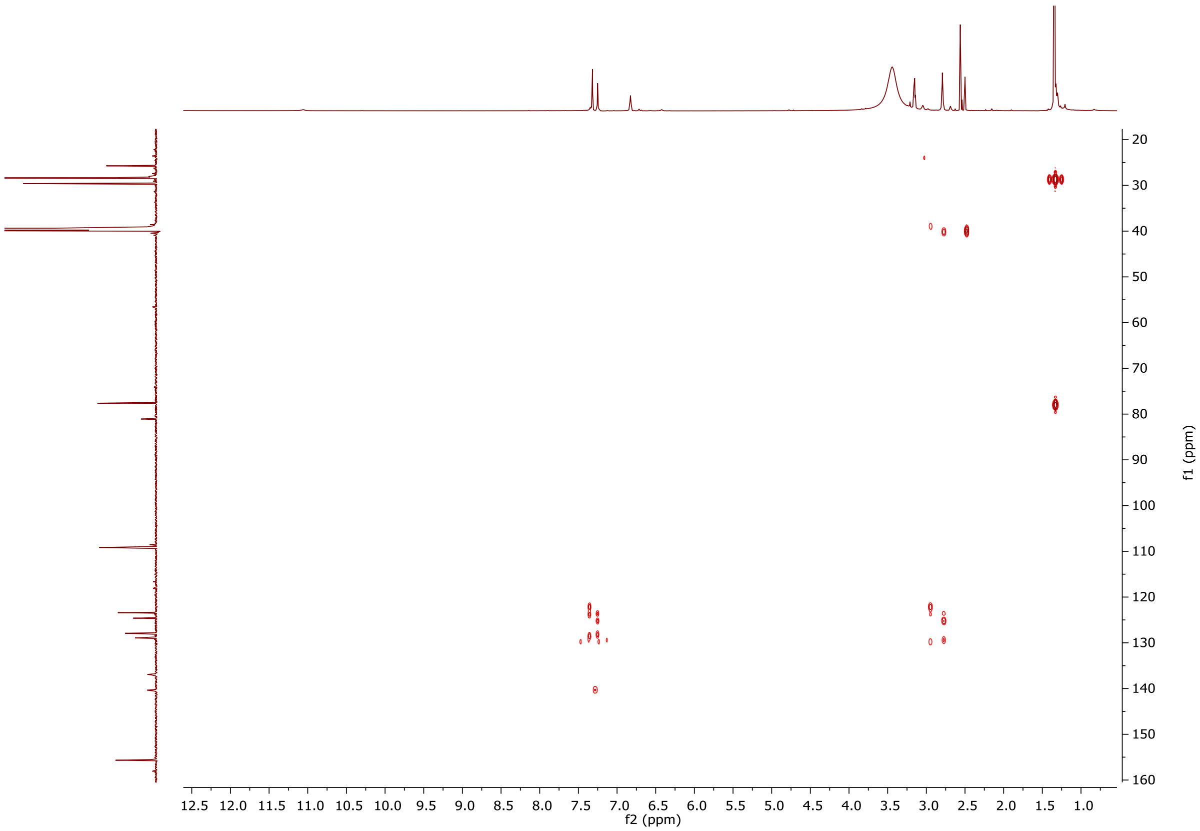

S26: ^{1}H NMR Spectrum for Compound **8** in DMSO-d_6

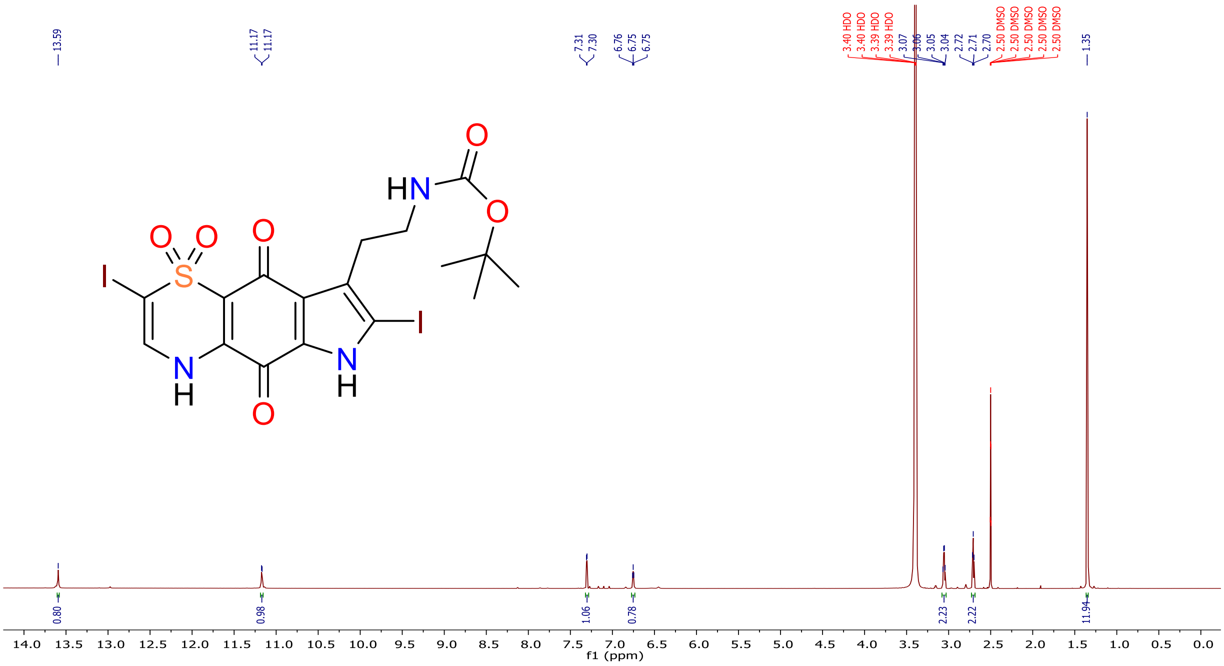

S27: ^{13}C NMR Spectrum for Compound **8** in DMSO-d_6

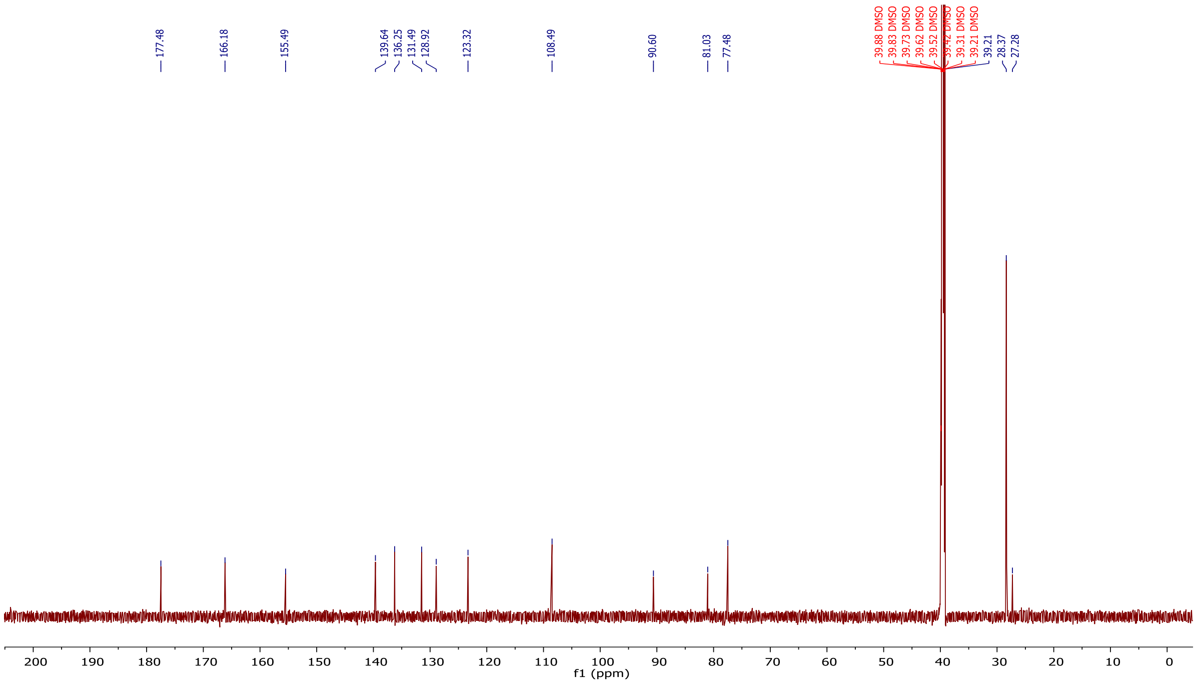

S28: COSY NMR Spectrum for Compound **8** in DMSO-d_6

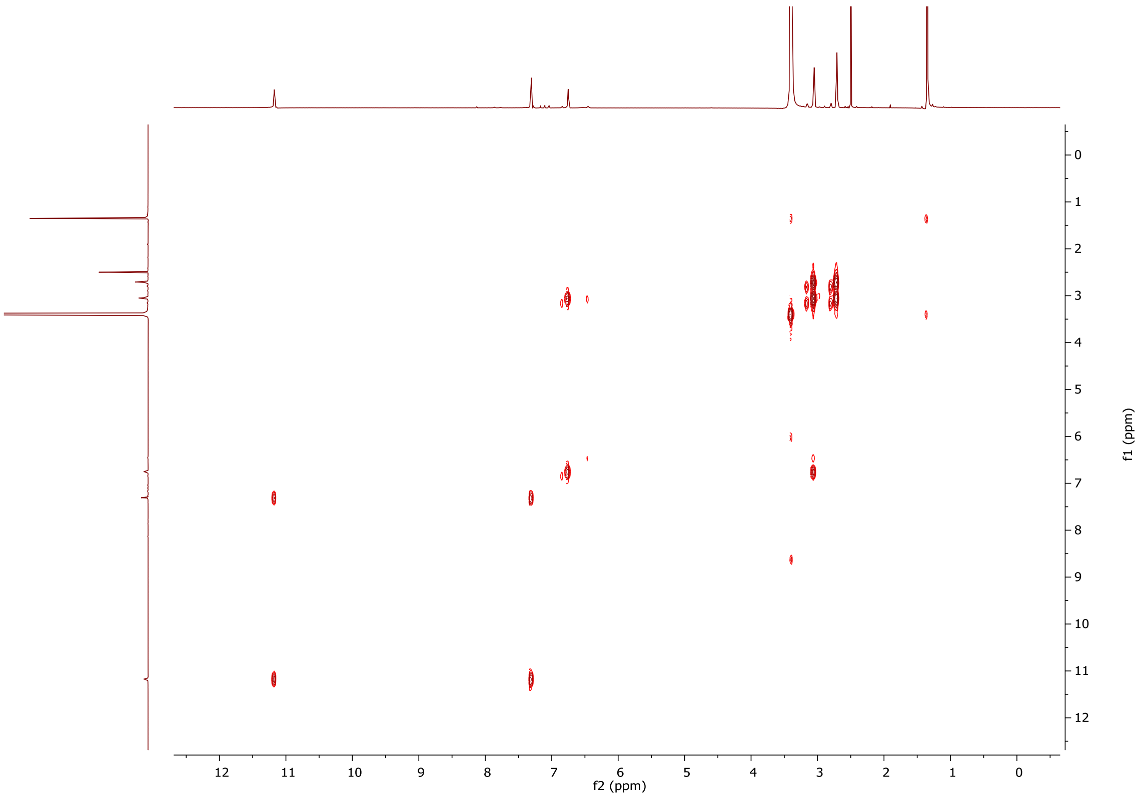

S29: HSQC NMR Spectrum for Compound **8** in DMSO-d_6

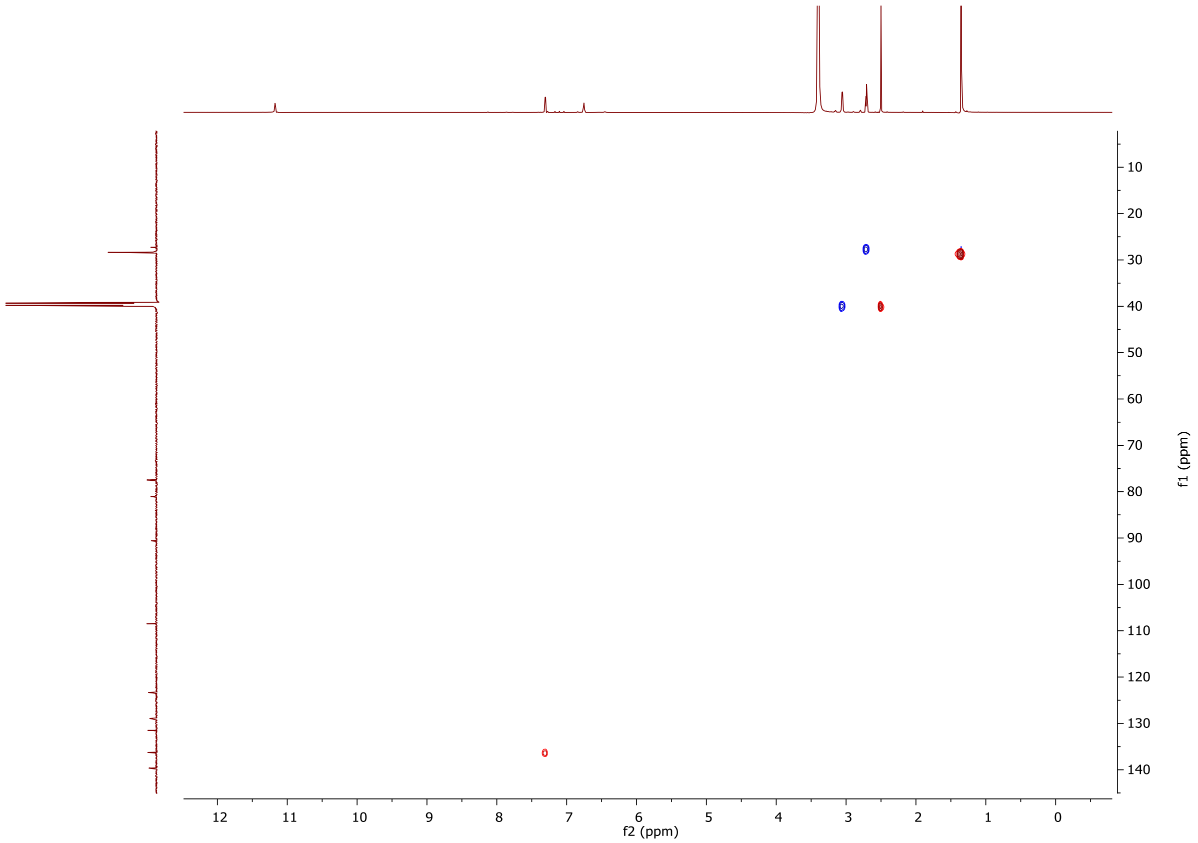

S30: HMBC NMR Spectrum for Compound **8** in DMSO-d_6

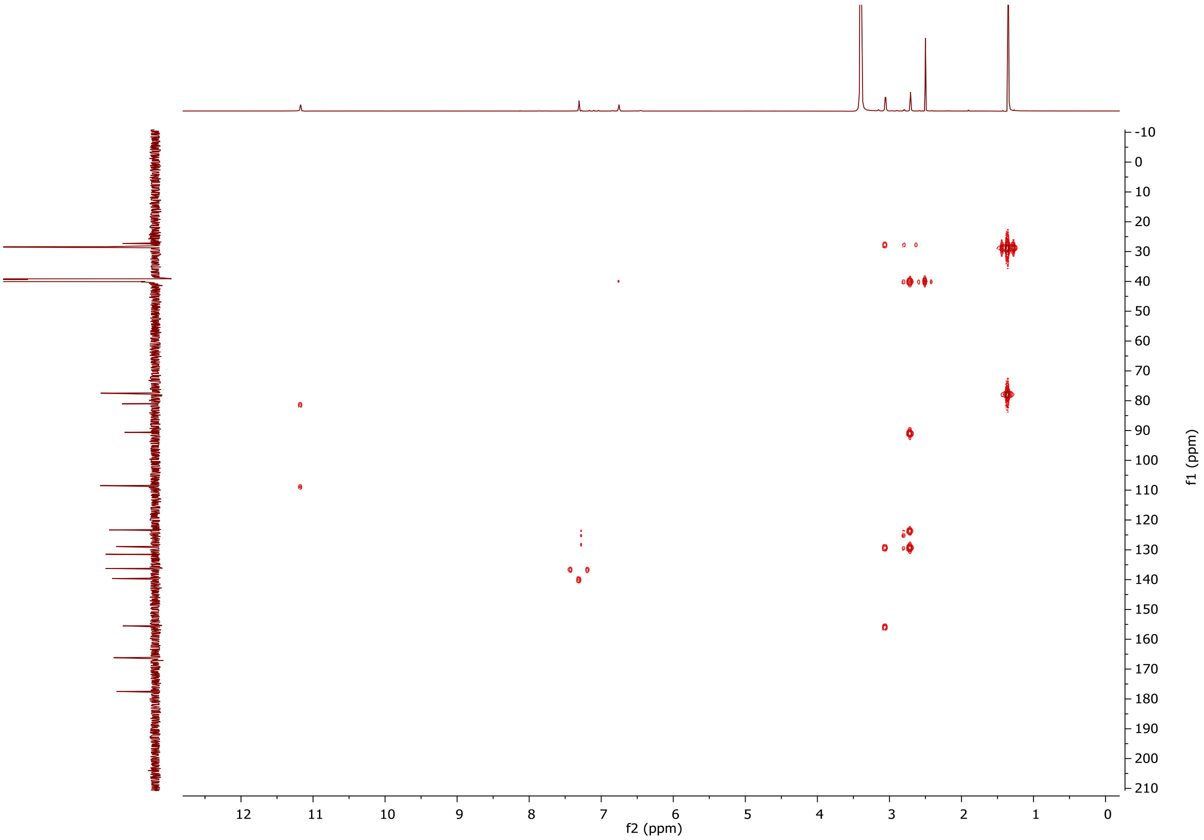

S31: ^{1}H NMR Spectrum for Compound **9** in DMSO-d_6

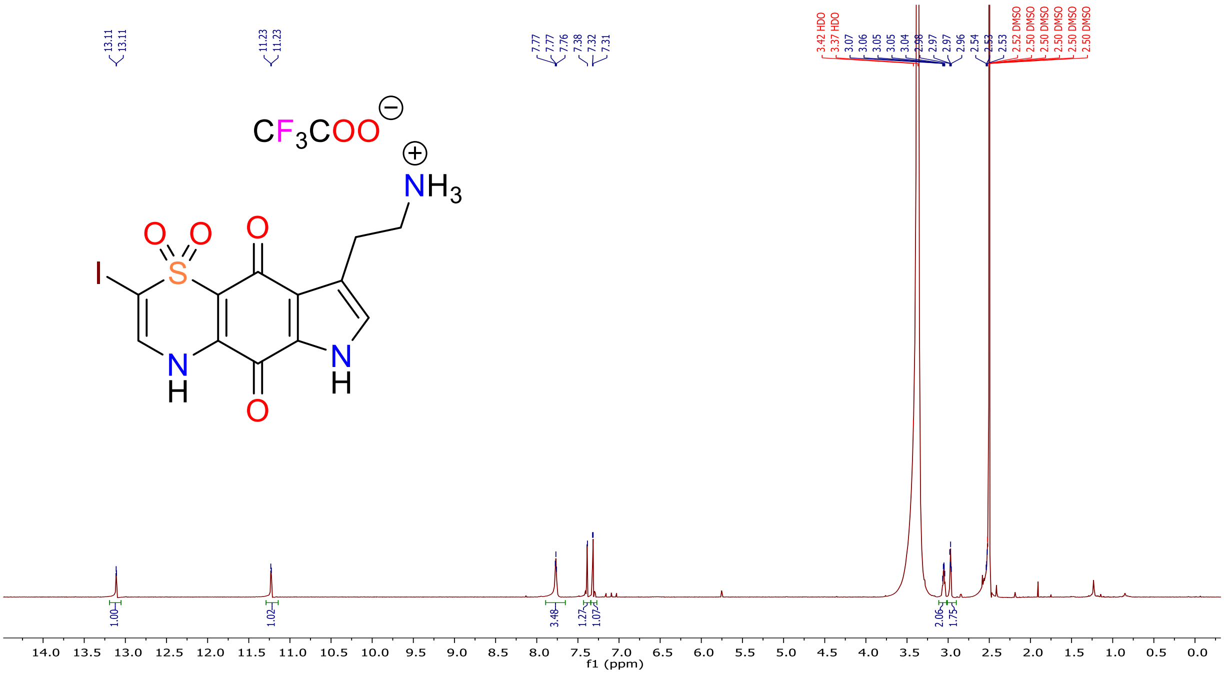

S32: ^{13}C NMR Spectrum for Compound **9** in DMSO-d_6

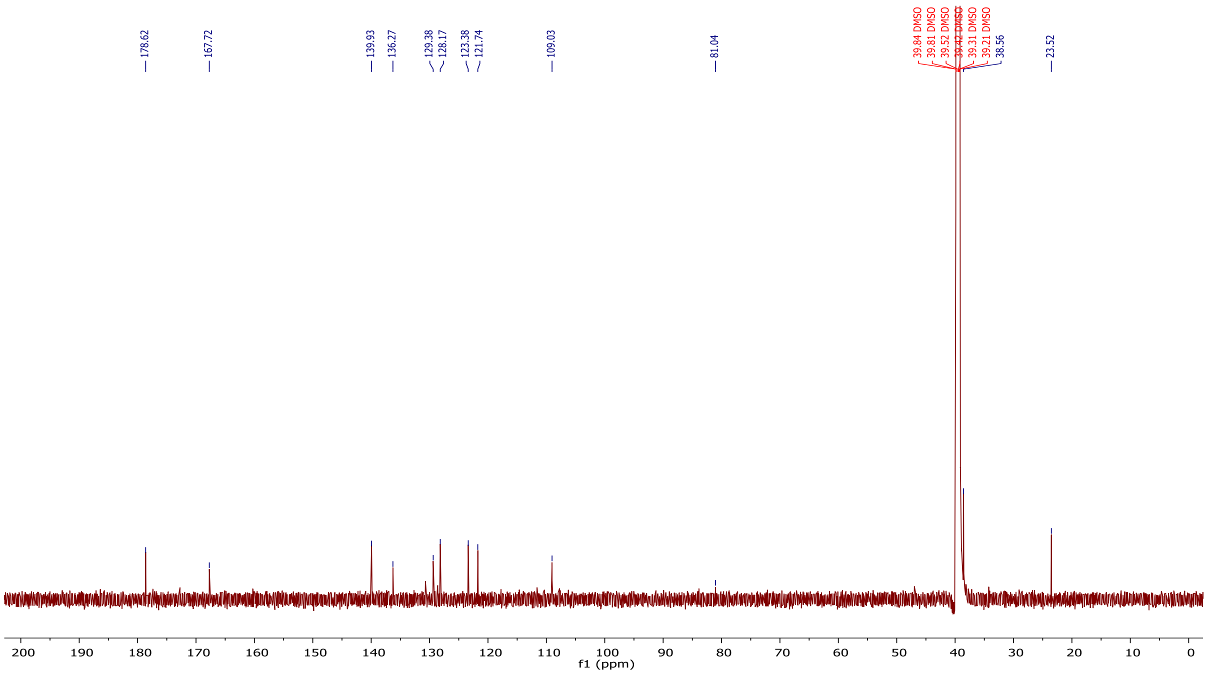

S33: COSY NMR Spectrum for Compound **9** in DMSO-d_6

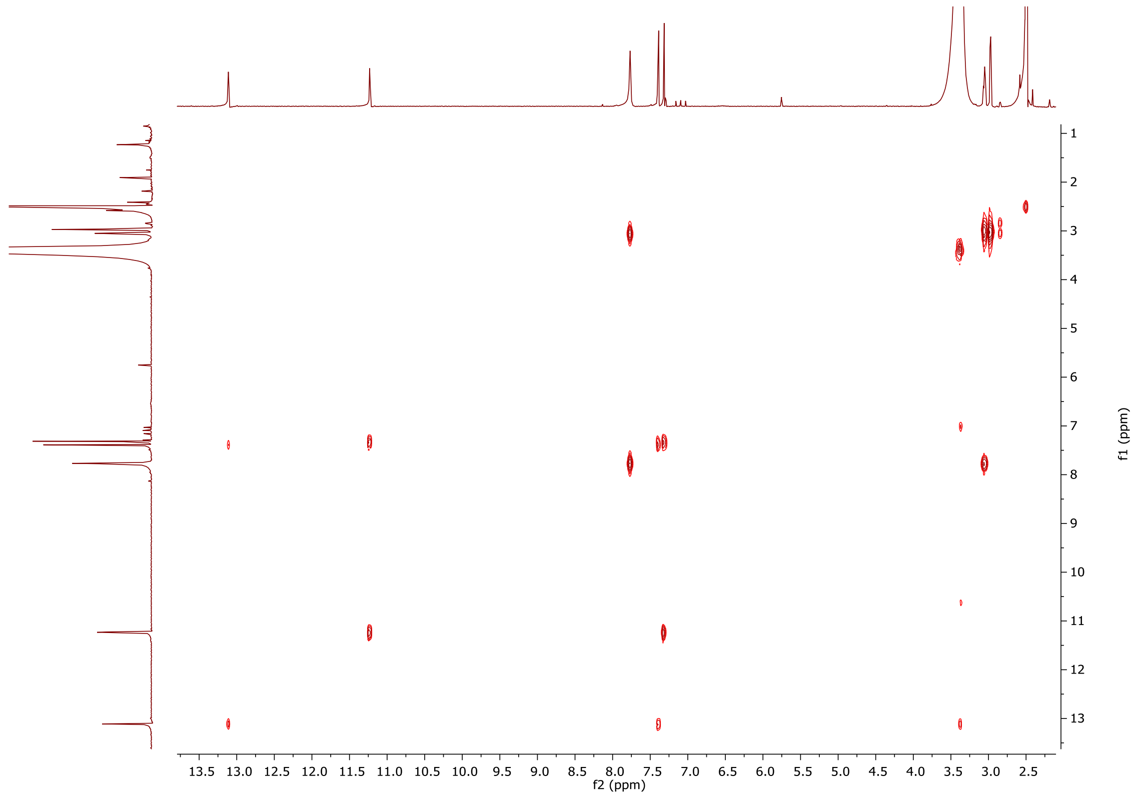

S34: HSQC NMR Spectrum for Compound **9** in DMSO-d_6

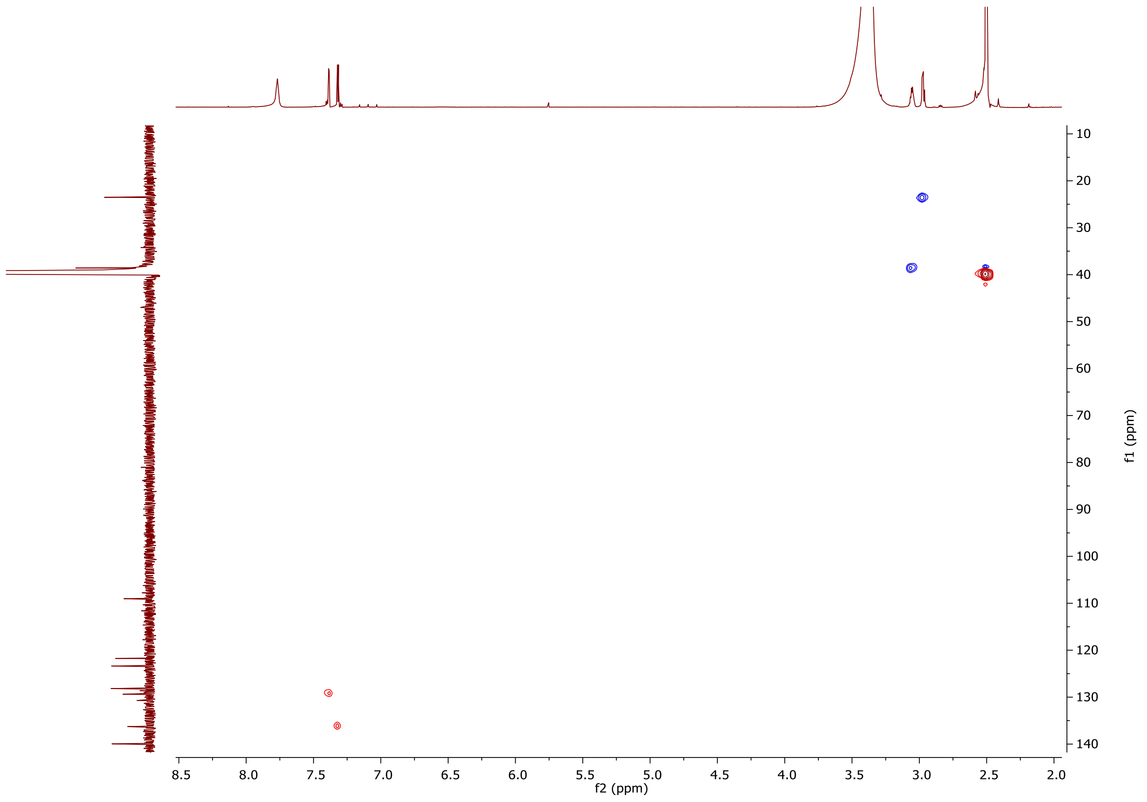

S35: HMBC NMR Spectrum for Compound **9** in DMSO-d_6

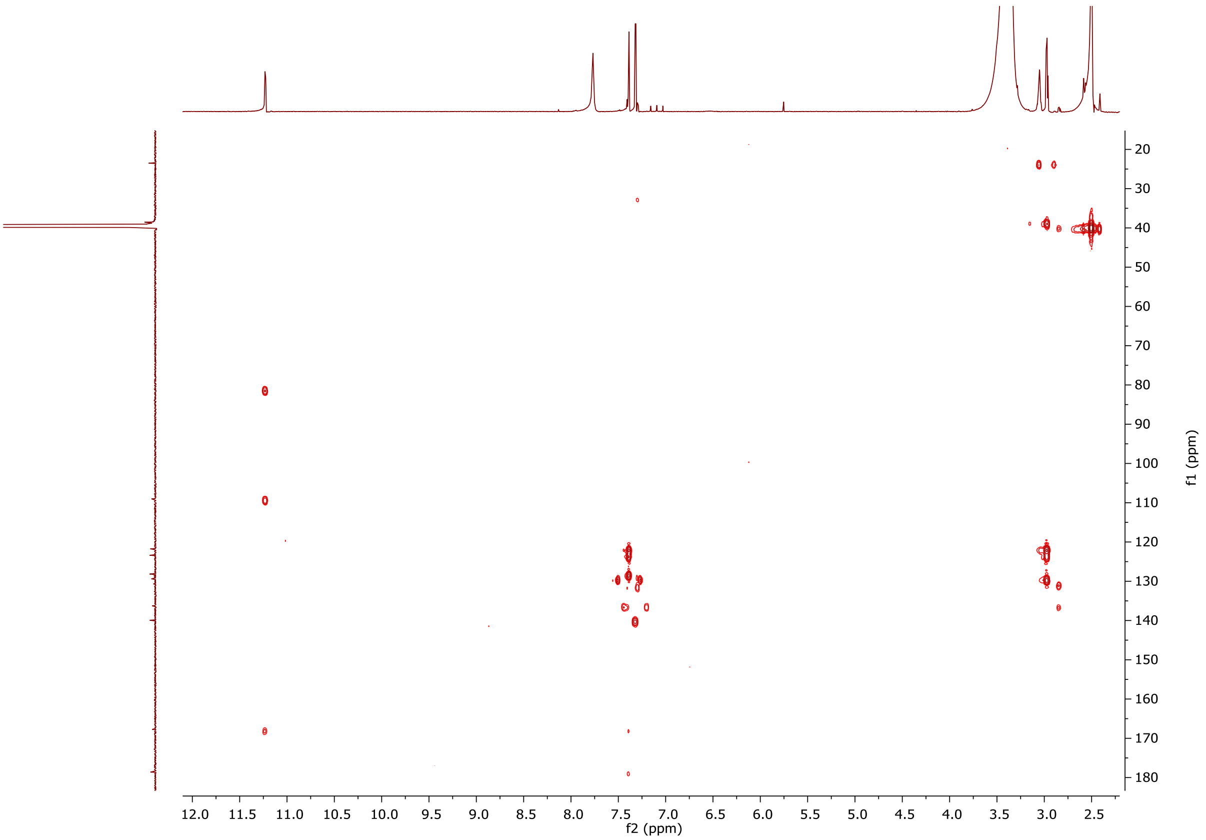

S36: ^{1}H NMR Spectrum for Compound **10** in DMSO-d_6

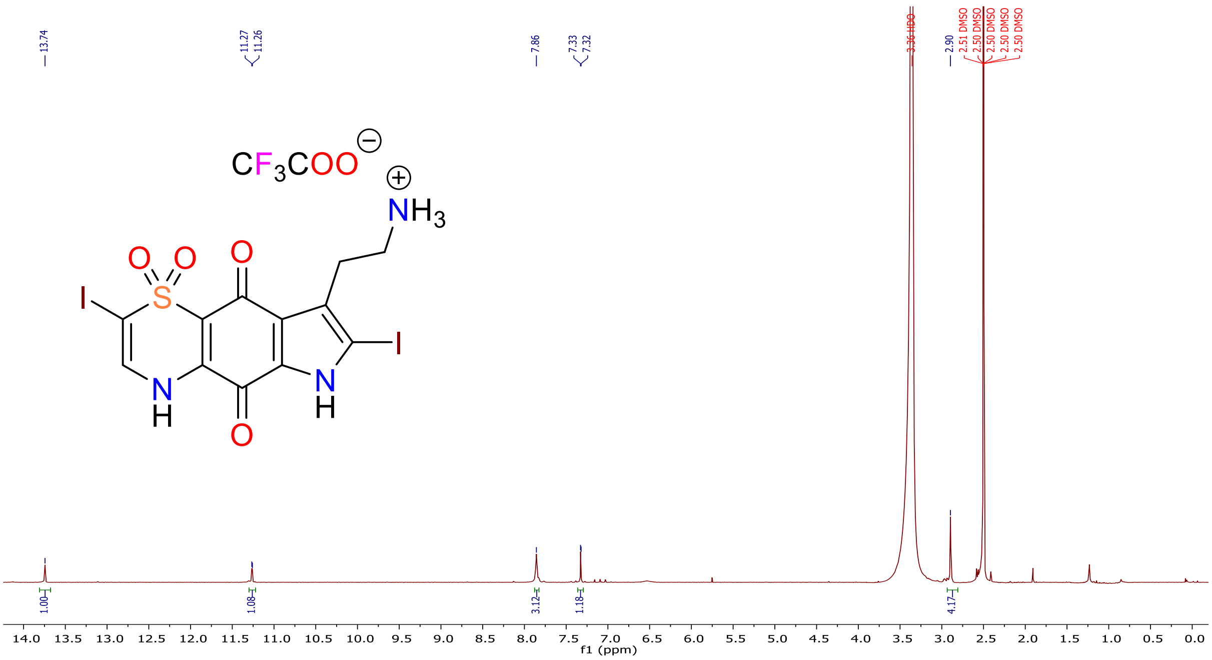

S37: ^{13}C NMR Spectrum for Compound **10** in DMSO-d_6

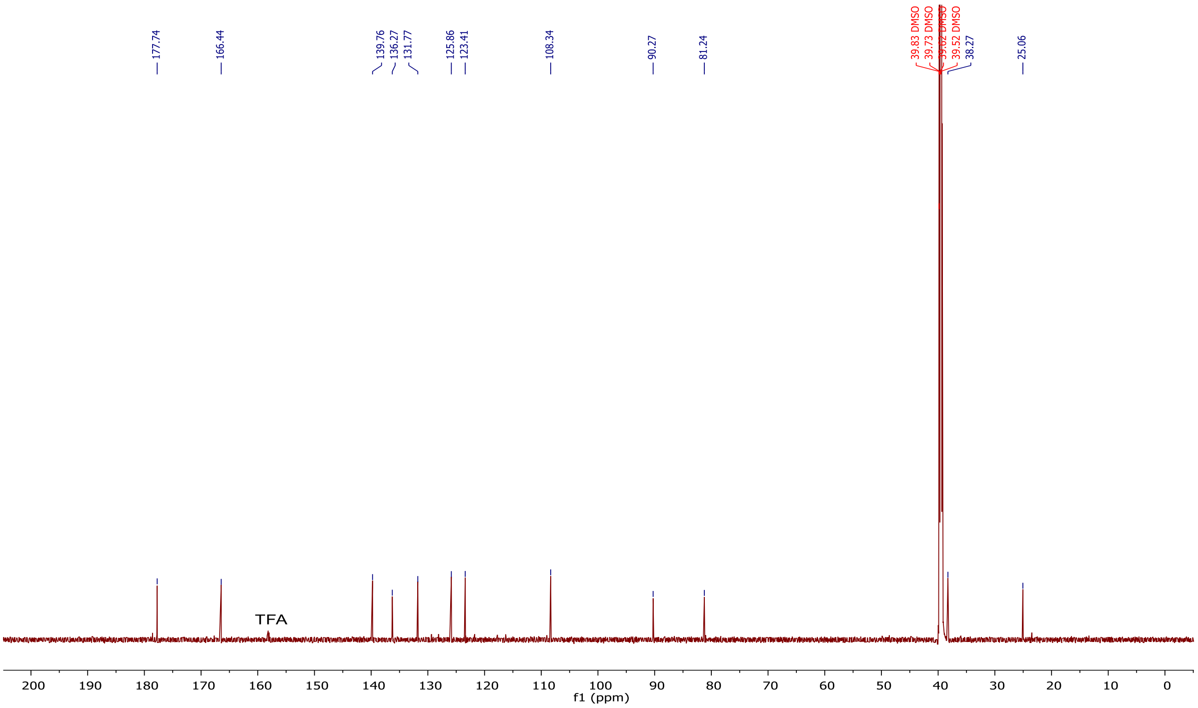

S38: COSY NMR Spectrum for Compound **10** in DMSO-d_6

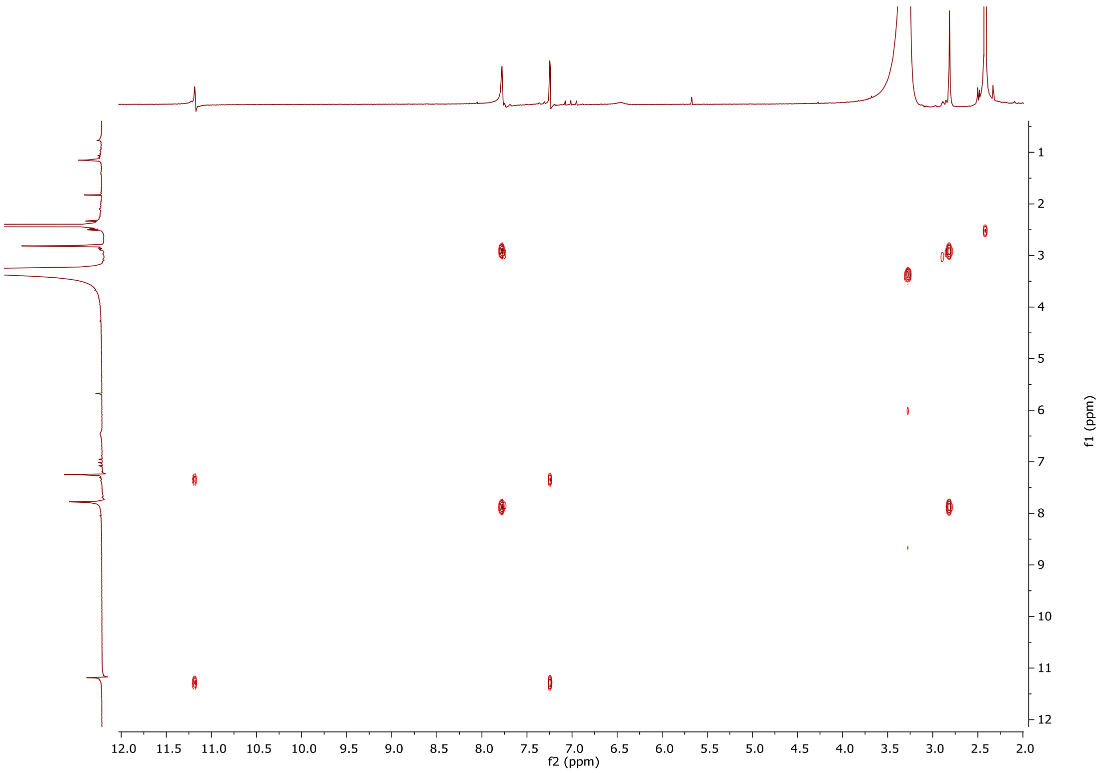

S39: HSQC NMR Spectrum for Compound **10** in DMSO-d_6

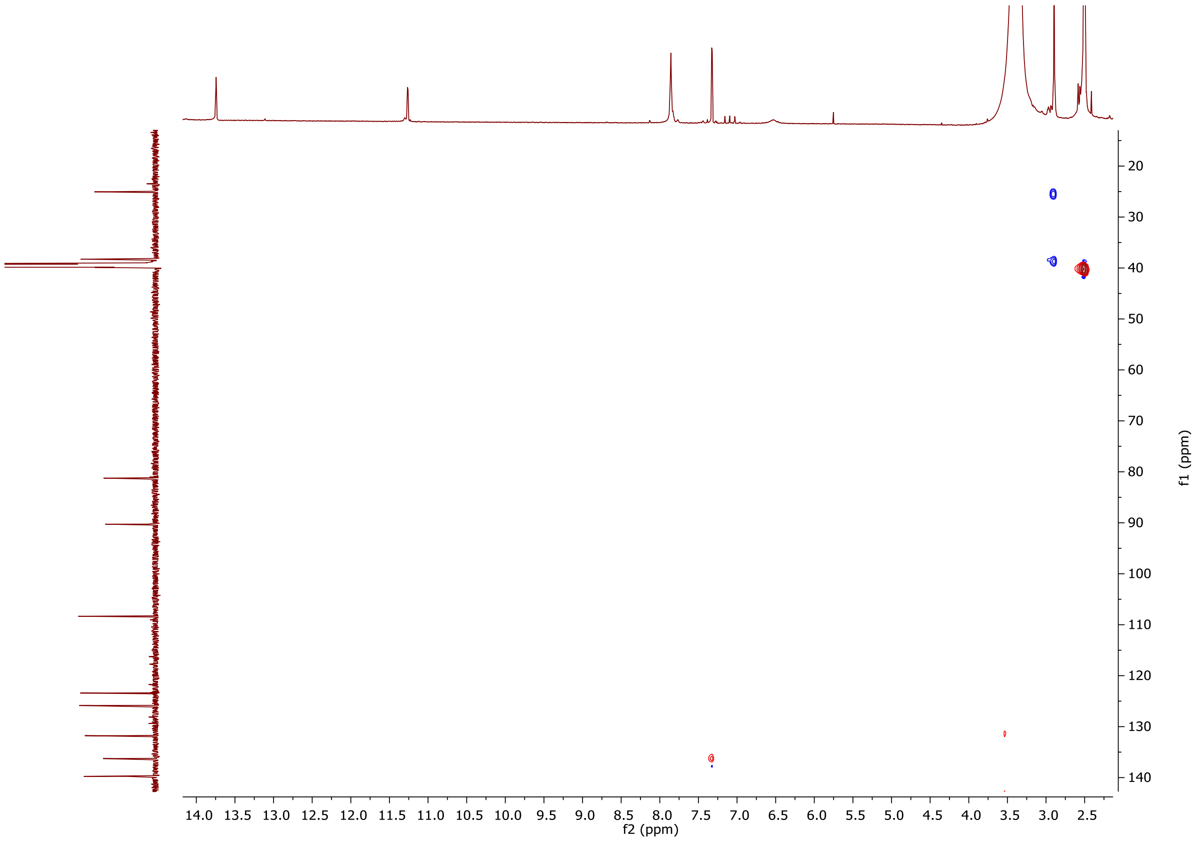

S40: HMBC NMR Spectrum for Compound **10** in DMSO-d_6

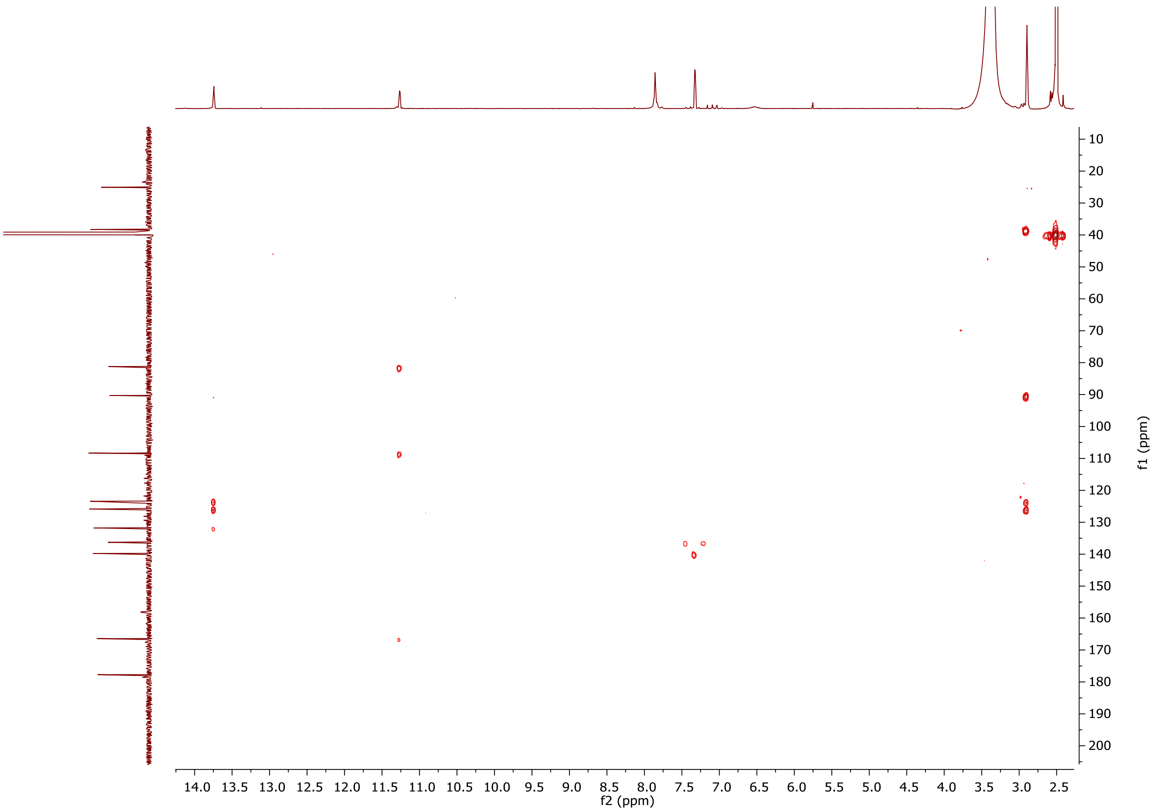

S41: ^{1}H NMR Spectrum for Compound **11** in DMSO-d_6

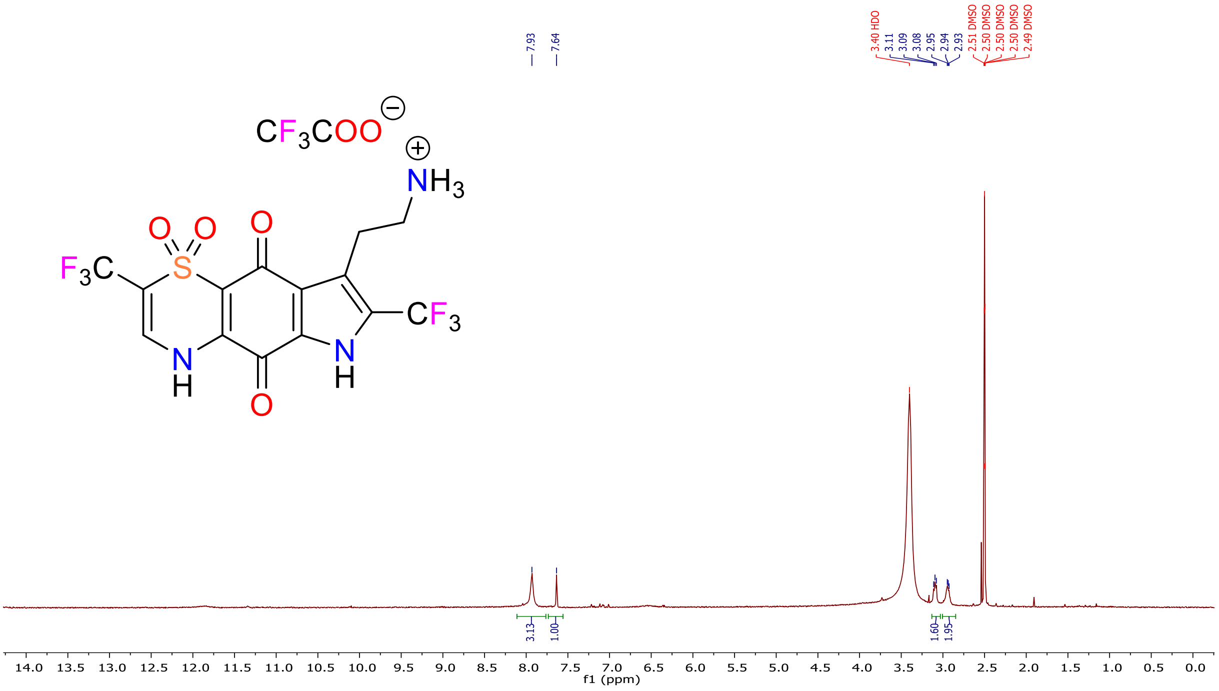

S42: ^{13}C NMR Spectrum for Compound **11** in DMSO-d_6

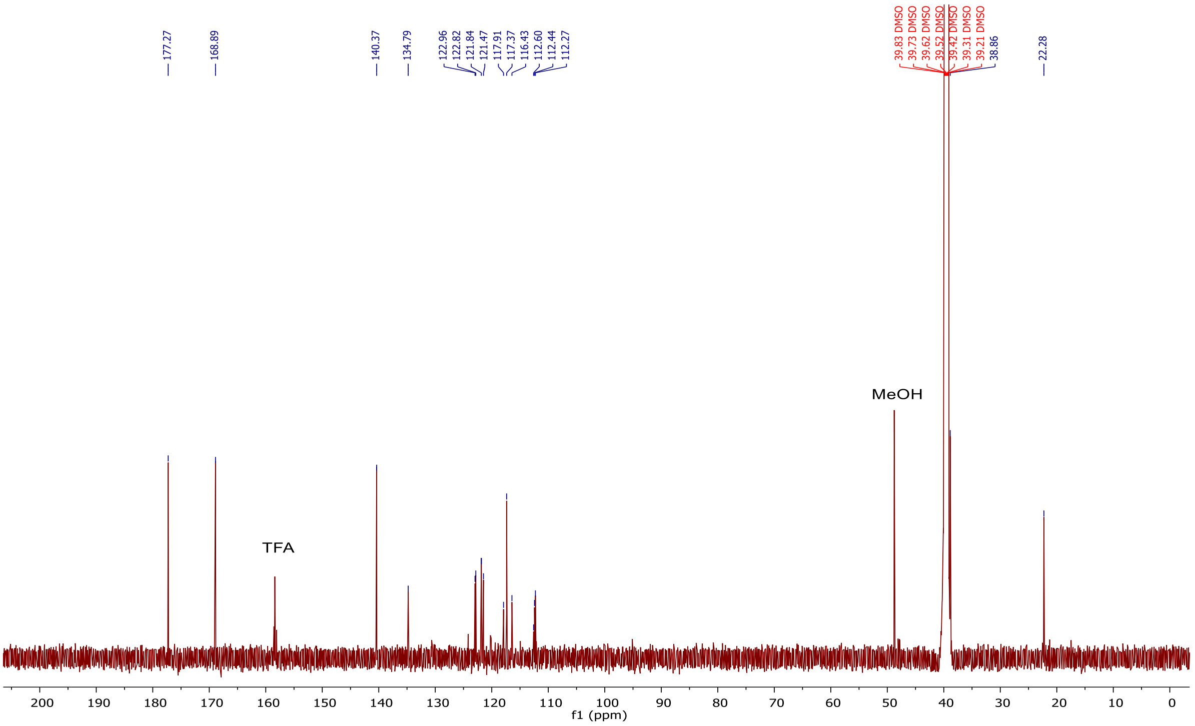

S43: COSY NMR Spectrum for Compound **11** in DMSO-d_6

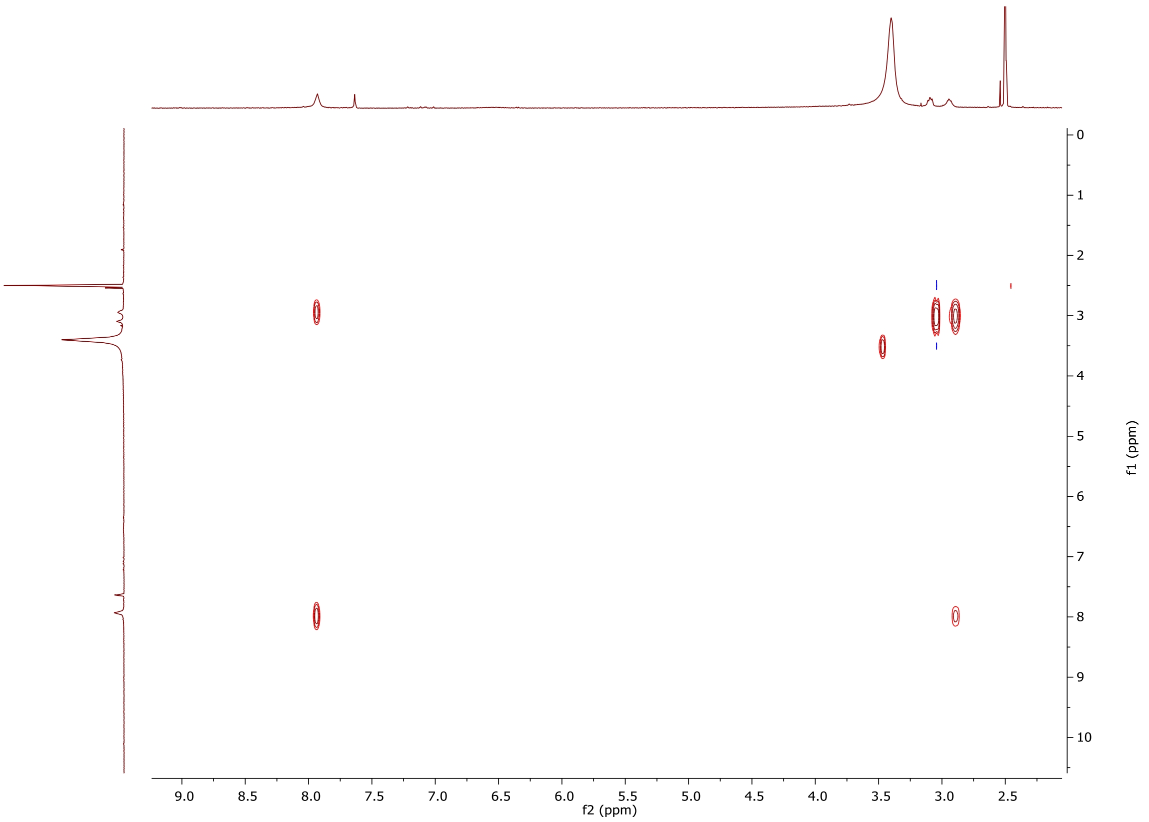

S44: HSQC NMR Spectrum for Compound **11** in DMSO-d_6

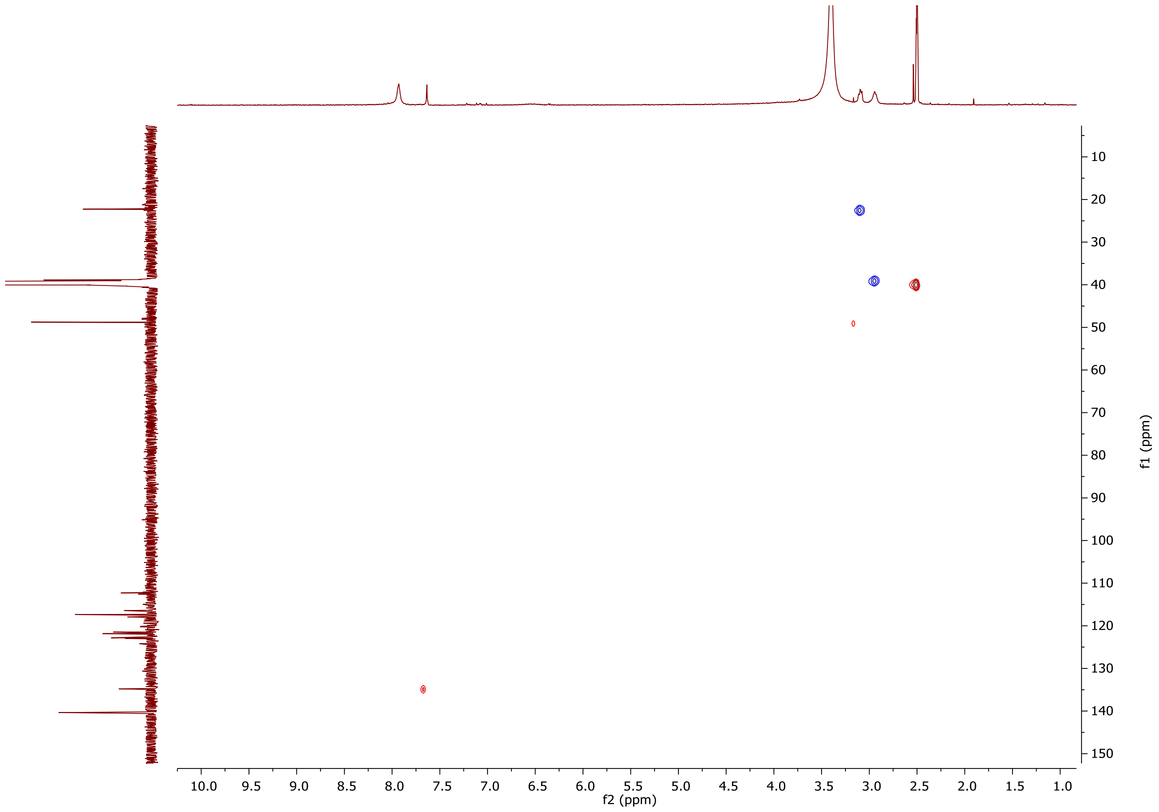

S45: HMBC NMR Spectrum for Compound **11** in DMSO-d_6

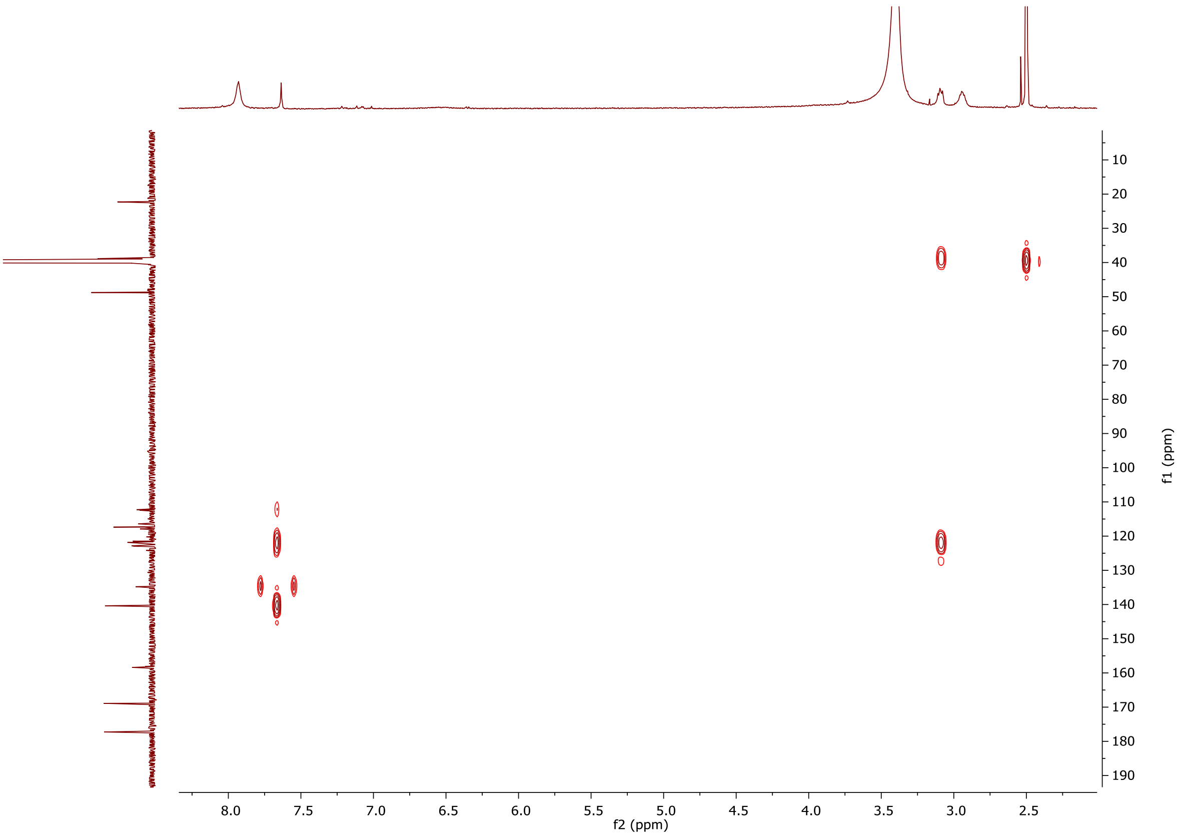

S46: Synthetic Route for Thiaplakortone A (HCl Salt)[1]

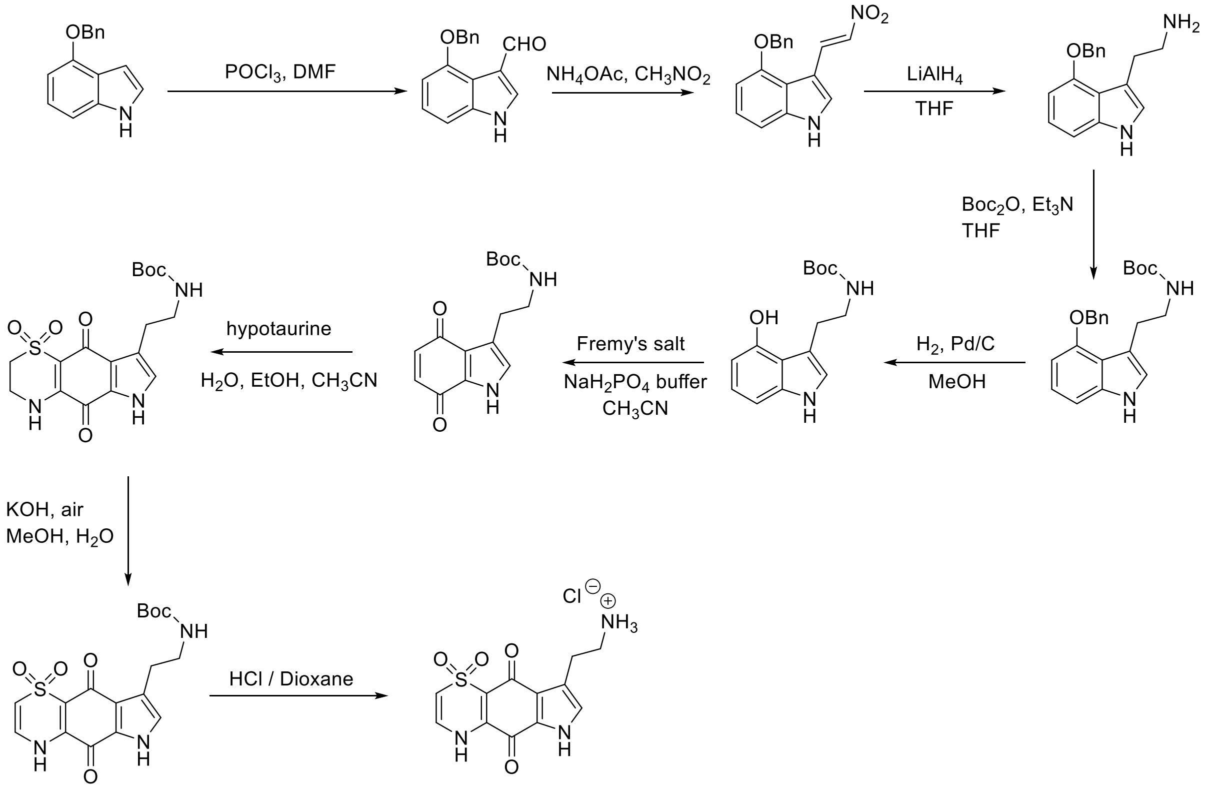

Thiaplakortone A (1)

1. Pouwer, R. H.; Deydier, S. M.; Le, P. V.; Schwartz, B. D.; Franken, N. C.; Davis, R. A.; Coster, M. J.; Charman, S. A.; Edstein, M. D.; Skinner-Adams, T. S.; Andrews, K. T.; Jenkins, I. D.; J. Quinn, R. J. Total synthesis of thiaplakortone A: derivatives as metabolically stable leads for the treatment of malaria. *ACS Med. Chem. Lett.* **2014**, *5*, 178–182.
